# New Bidirectional Ammonia Flux Model in an Air Quality Model Coupled With an Agricultural Model

**DOI:** 10.1029/2019MS001728

**Published:** 2019-10-24

**Authors:** Jonathan E. Pleim, Limei Ran, Wyat Appel, Mark W. Shephard, Karen Cady-Pereira

**Affiliations:** 1 U.S. Environmental Protection Agency, Research Triangle Park, NC, USA; 2 Environment and Climate Change Canada, Toronto, Ontario, Canada; 3 Atmospheric and Environmental Research, Inc., Lexington, MA, USA

## Abstract

Ammonia surface flux is bidirectional; that is, net flux can be either upward or downward. In fertilized agricultural croplands and grasslands there is usually more emission than deposition especially in midday during warmer seasons. In North America, most of the ammonia emissions are from agriculture with a significant fraction of that coming from fertilizer. A new bidirectional ammonia flux modeling system has been developed in the Community Multiscale Air Quality (CMAQ) model, which has close linkages with the Environmental Policy Integrated Climate (EPIC) agricultural ecosystem model. Daily inputs from EPIC are used to calculate soil ammonia concentrations that are combined with air concentrations in CMAQ to calculate bidirectional surface flux. The model is evaluated against surface measurements of NH_3_ concentrations, NH_4_^+^ and SO_4_^2−^ aerosol concentrations, NH_4_^+^ wet deposition measurements, and satellite retrievals of NH_3_ concentrations. The evaluation shows significant improvement over the base model without bidirectional ammonia flux. Comparisons to monthly average satellite retrievals show similar spatial distribution with the highest ammonia concentrations in the Central Valley of California (CA), the Snake River valley in Idaho, and the western High Plains. In most areas the model underestimates, but in the Central Valley of CA, it generally overestimates ammonia concentration. Case study analyses indicate that modeled high fluxes of ammonia in CA are often caused by anomalous high soil ammonia loading from EPIC for particular crop types. While further improvements to parameterizations in EPIC and CMAQ are recommended, this system is a significant advance over previous ammonia bidirectional surface flux models.

## Plain Language Summary

Ammonia is one of many pollutants that are added to the air by human activities. Ammonia contributes to degraded air quality mainly because it adds to the concentration of very fine particles suspended in the air that are called aerosols. Hazy conditions with poor visibility are often the result of high concentrations of aerosols. Also, breathing air with high amounts of aerosols is bad for human health. Ammonia can get into water bodies where it contributes to algae growth and fish kills due to low oxygen. Much of the ammonia that gets into the air comes from fertilizer in agricultural fields. This paper is about improved methods for modeling the ammonia from agricultural fertilizer by linking together an air quality computer model with an agricultural management model. The agricultural model predicts the amount and types of fertilizer that farmers use and the amount of ammonia in the soil. The air quality model then uses information from the agriculture model to predict how much ammonia gets into the air. The study describes these models and shows how well they predict the amount of ammonia in the air compared to ground-based and satellite measurements.

## 1. Introduction

Inorganic reduced forms of reactive nitrogen (N; e.g., ammonia NH_3_ and ammonium NH_4_^+^) are important precursors to fine particulate matter (PM_2.5_), which is known to be a serious human health hazard (Anderson et al., 2012; Kim et al., 2015) and is subject to regulation through the National Ambient Air Quality Standards (NAAQS). About 30% of total NH_3_ emissions in the United States come from fertilization of agricultural grass and crop production and 54% come from animal husbandry (Xing et al., 2013) based on the U.S. Environmental Protection Agency (EPA) 2002 National Emissions Inventory (NEI). Most of N applied to agricultural lands are in the form of synthetic fertilizer. In addition to contributing to the atmospheric aerosol burden, N also runs off fields into other ecosystems and water bodies where it contributes to environmental degradation through eutrophication, acidification, and loss of biodiversity (Boyle, 2017; Phoenix et al., 2012; Stevens et al., 2004).

There are a range of modeling approaches to estimate ammonia emissions from fertilization, which vary greatly in their complexity and data requirements. A common approach is to use emission factors multiplied by fertilizer sales data with simple seasonal functions. For example, the 2011 U.S. EPA NEI calculates ammonia emissions using the Carnegie Mellon University Ammonia Model v.3.6 (Goebes et al., 2003), which distributes county-level fertilizer sales to agricultural land on a monthly basis (U.S. EPA, 2015). Balasubramanian et al. (2015) describe an alternative method that also uses fertilizer sales data but with improved spatial representation from high resolution crop data and daily temporal information from the Denitrification Decomposition (DNDC) model. In addition, Hamaoui-Laguel et al. (2014) developed a 1-D mechanistical model for estimating ammonia emissions from fertilized agricultural fields with management practices, meteorology, and soil property information. In recent years some air quality models have transitioned from emission factor parameterizations for NH_3_ emissions to more complex process-based models including algorithms for bidirectional surface flux of NH_3_ particularly from fertilized agriculture land. Rather than using separate estimates of NH_3_ emissions from an emission inventory combined with dry deposition (i.e. one-way flux from air to surface), bidirectional algorithms obtain estimates of the compensation concentrations in the surface, primarily soil, and leaf tissue, then compute the net NH_3_ fluxes as functions of concentration difference between air and surface (Massad et al., 2010). A key issue for bidirectional flux models is the specification of the ammonia concentrations in the surface components such as soil and stomata. At the air-surface interface inside leaf stomatal cavities and in soil pores, gas-phase ammonia concentrations can be estimated by applying Henry’s law and thus requiring equilibrium with aqueous concentrations of NH_4_^+^ and H^+^ in the apoplast leaf tissues and soil water (Sutton et al., 2000). Air NH_3_ concentrations are computed from the ammonia emission potential *Γ*, which is the ratio of NH_4_^+^ to H^+^.

Ammonia bidirectional flux models have recently been applied in several regional and global atmospheric chemistry models. For example, Zhang et al. (2010) have implemented a bidirectional flux model for NH_3_, which uses a resistance approach similar to dry deposition models in the Canadian air quality model Global Environmental Multi-scale-Modelling air quality and Chemistry (GEM-MACH; Whaley et al., 2018). In this system, ammonia emission potentials for soil and stomata (*Γ*_*g*_ and *Γ*_*st*_,), specified by land use category, were empirically derived from an extensive review of observational studies in a variety of environments. While this approach captures spatial variations due to land use, the *Γ*_*g*_ and *Γ*_*st*_ values are constant so they do not respond to timing and rate of fertilization. Massad et al. (2010) describe a similar approach where ammonia emission potential is related to land use. However, their model considers the increases in *Γ*_*st*_ and *Γ*_*g*_ associated with fertilizer application followed by an exponential decay back to background levels. While this approach adds parameterized dynamics for response to fertilization, it requires additional input for fertilizer application amount, type, and timing.

Some process-based models include dynamic estimation of N concentrations in the soil and plant tissues. For example, Riddick et al. (2016) describe a highly parameterized ammonia emission model for the Community Land Model (CLM) that tracks the flow of agricultural N from both manure and synthetic fertilizer and includes parameterizations for runoff, nitrification, denitrification, soil diffusion, and volatilization. The NH_3_ compensation concentration is computed from the total ammoniacal nitrogen pool in the soil. For timing of fertilizer application, the CLM uses the spring planting date for corn from the CLM4.5 internal crop model (Levis et al., 2012). Zhu et al. (2015) describe a similar bidirectional ammonia flux model with parameterized soil ammonia pool that has been recently applied to the GEOS-Chem global atmospheric chemistry model. The soil N pool includes sources and sinks from wet and dry deposition, nitrification, volatilization, and fertilizer application. The effects of soil nitrification are parameterized by an exponential depletion of soil N assuming a 15-day time scale. Daily resolved fertilizer application rate is derived from annual total fertilization apportioned to the growing season using the Moderate Resolution Imaging Spectroradiometer (MODIS) enhanced vegetation index (Hudman et al., 2012). A drawback of these types of parameterized process models is that they include many uncertain parameters that are largely unconstrained by observations. Thus, while such models may include the major sources and sinks affecting the total ammoniacal nitrogen pool, the results may have greater uncertainty than direct emission factor approaches.

A more holistic approach is to use a process-based agricultural production model, which has key soil biogeochemical processes, hydrology, and management practices, including fertilization, and is driven by atmospheric conditions (e.g., meteorology and nitrogen deposition) consistent with air quality simulations. As N fertilization varies by type, rate, and timing, depending on production types and geographical area, the agricultural model can resolve soil physical and chemical properties on managed land much more realistically in time and space with different soil and micrometeorology conditions. Simulated soil ammonia concentration along with soil physical properties from the agricultural model can be directly input to bidirectional NH_3_ modeling. There are many cropping systems (e.g., DSSAT (Jones et al., 2003), DNDC (Li, 2000), EPIC (Williams, 1995), and STICS (Brisson et al., 2003)) developed over the years and regularly applied worldwide in research and assessment studies. These systems differ in model structure and approach to representation of complicated physical and biogeochemical processes (Brilli et al., 2017. The Environmental Policy Integrated Climate (EPIC) model is an agricultural ecosystem model, which has been developed with support from the U.S. Department of Agriculture (USDA, https://epicapex.tamu.edu/epic/) since the early 1980s. The system is a comprehensive terrestrial model with key physical and biogeochemical processes simulating plant growth, water and carbon balance, soil erosion, and nutrient cycling (e.g., N and phosphorus) under the influence of soil, landscape, weather, climate, and management conditions (Williams, 1995; Williams et al., 1984). The EPIC model has been extensively evaluated for many cropping systems across the world and used by USDA assessment projects to examine impacts of management practices and climate on agricultural production, soil productivity, and nutrient runoff (Benson et al., 1989; Rosenzweig et al., 2014; White et al., 2014).

Cooter et al. (2010) showed that bidirectional NH_3_ flux from managed agricultural soils can be reasonably estimated using a resistance and compensation point flux approach (Nemitz et al., 2000) and the deposition model (Pleim & Ran, 2011) from the Community Multiscale Air Quality model (CMAQ, https://www.epa.gov/cmaq; Byun & Schere, 2006) integrated with components of EPIC. Based on their initial results, the Fertilizer Emission Scenario Tool for CMAQ (FEST-C, https://www.cmascenter.org/fest-c/; Ran et al., 2011; Cooter et al., 2012) was first developed to integrate EPIC with a meteorology and air quality modeling system—Weather Research and Forecast model (WRF, http://www2.mmm.ucar.edu/wrf/users/; Skamarock et al., 2008) and CMAQ (WRF-CMAQ). Using WRF-CMAQ weather and N deposition, the system facilitates EPIC simulations for 42 different production types, with management practices corresponding to different agricultural production regions across the conterminous U.S. (CONUS). FEST-C can extract soil chemistry with N fertilizer information on agricultural land from EPIC results, thus generating the necessary inputs for CMAQ bidirectional NH_3_ flux modeling (Cooter et al., 2012). The FEST-C system is released with an updated version of EPIC, which is adapted to regional-scale applications for CONUS. With the continuous support from U.S. EPA, FEST-C has now been enhanced by integrating the Soil and Water Assessment Tool (SWAT, https://swat.tamu.edu/) modeling system (Arnold et al., 1998) with EPIC and WRF-CMAQ for improving our understanding of agricultural production, weather, and N deposition impacts on hydrology and water quality for large river basins (Yuan et al., 2018). A detailed description and assessment of the recent release of FEST-C V1.4 will be presented in an upcoming publication. With its multiple capabilities, the system is now a valuable tool in integrated modeling for assessing interactions among land-water-air multimedia processes.

The focus of this paper is on the description and evaluation of a new implementation of bidirectional ammonia flux modeling in CMAQ coupled directly with FEST-C EPIC for soil characteristics including NH_3_ content. Section 2 includes brief descriptions of the WRF, CMAQ, and FEST-C systems along with detailed description of the bidirectional ammonia flux algorithm in CMAQ, focusing on recent developments. Evaluations of resulting NH_3_ gas and NH_4_^+^ and SO_4_^2−^ aerosol air concentrations and NH_4_^+^ wet deposition compared to surface measurements and satellite retrievals are described in section 3. Section 4 provides further discussion of the evaluation, including in-depth analyses of case studies where the evaluation shows particularly large discrepancies between model and measurements. Concluding remarks are given in section 5.

## 2. Model Description

A bidirectional NH_3_ surface flux model was developed for application in regional to global air quality models and evaluated in box-model form compared to agricultural field measurements (Walker et al., 2013, 2006) as described by Pleim et al. (2013). This study demonstrated that the bidirectional flux model can capture the magnitude and dynamics of measured ammonia fluxes over a range of conditions with overall biases on the order of the uncertainty of the measurements when using soil chemistry and moisture provided from simulations by the EPIC agricultural model (Williams, 1995). The bidirectional NH_3_ flux model was then incorporated and tested in the WRF-CMAQ coupled meteorology and air quality model (Bash et al., 2013). However, the implementation described by Bash et al. (2013) differed from the box model study in the way the soil chemistry was estimated. Rather than using soil ammonia content daily directly from EPIC, the previous CMAQ implementation used EPIC simulated soil ammonia content only for initial conditions and then estimated changes to soil ammonia content using EPIC-simulated daily fertilization information following Massad et al. (2010). The estimation of soil ammonia concentration also included CMAQ computed evasion and deposition and EPIC nitrification algorithms adapted to CMAQ. However, other significant N pathways such as fixation, mineralization, denitrification, runoff, percolation, and plant uptake, which are fully accounted for in EPIC, are missing from the previous CMAQ implementation (Cooter et al., 2012). Thus, a drawback to this method is that the soil ammonia concentrations would quickly diverge from the EPIC model simulations. Consequently, CMAQ simulations of ammonia concentrations would differ depending on initialization dates (Hogrefe et al., 2017) and meteorology conditions.

In this paper we describe a new implementation of bidirectional flux modeling that follows directly from the box model demonstration presented by Pleim et al. (2013). The modeling system used in this study includes EPIC, WRF, and CMAQ. Since this study focuses on modeling surface fluxes of ammonia, the new and revised components of the system related to these processes are described.

### 2.1. The PX LSM and CMAQ Dry Deposition Model

The CMAQ model is a regional-to-local scale chemical transport model that can be either coupled to the WRF meteorology model (Wong et al., 2012) or run sequentially using meteorological input from the WRF model. The NH_3_ bidirectional flux model is developed as an adjunct to the CMAQ dry deposition model (Pleim & Ran, 2011). The CMAQ dry deposition model (also known as M3DRY) is designed to be consistent with the PX land surface model (PX LSM), which is one of the LSM options in the WRF system (Pleim & Xiu, 1995; Xiu & Pleim, 2001). Thus, many of the parameters used in the dry deposition and bidirectional NH_3_ modeling in CMAQ are inherited from the surface moisture flux modeling in the WRF PX LSM. In addition, many of the key parameters for NH_3_ bidirectional surface flux model are obtained from model simulations of FEST-C EPIC as described below.

### 2.2. FEST-C EPIC

The CMAQ bidirectional NH_3_ flux calculations require inputs describing the physical properties of the soil, as well as NH_3_ content and agricultural land use data, all of which are generated using the FEST-C interface. The FEST-C system is available from the Community Modeling and Analysis System (CMAS) Center (https://www.cmascenter.org/) with the support from U.S. EPA. The released package is developed for Linux system applications and comes with a Java-based interface, an adapted implementation of EPIC for WRF-CMAQ regional grid domain applications, the required data sets, and built-in tools (Ran et al., 2018). The interface guides users through land use and EPIC input data generation, EPIC simulations with WRF-CMAQ weather and N deposition input, and the generation of inputs required for CMAQ bidirectional NH_3_ modeling from EPIC results. As EPIC is a field-scale model, FEST-C EPIC is adapted to CMAQ grid domains covering CONUS at different resolutions and in any of the four WRF projection coordinate systems: longitude/latitude, Lambert Conformal Conic, Universal Polar Stereographic, and Mercator. Detailed information on FEST-C EPIC and FEST-C interface tools is presented by Cooter et al. (2012) and the FEST-C user’s guide (Ran et al., 2018). There are many updates in FEST-C V1.4 including EPIC improvements and enhanced tools. Thus, FEST-C V1.4 is required for generating and extracting EPIC soil pH, porosity, wilting point, cation-exchange capacity, depth, moisture, and NH_3_-N concentration, which are needed by this new CMAQ-bidirectional NH_3_ flux model.

### 2.3. CMAQ NH_3_ Bidirectional Flux

#### 2.3.1. Calculation of Soil Ammonia From EPIC Input

After an extensive model spin-up period of typically 25 years (100 years for potatoes) using climatological meteorology data, the EPIC model is run for the specific year to be modeled by CMAQ with the same WRF meteorology as used to drive CMAQ on the same spatial grid. FEST-C EPIC also uses CMAQ N deposition input. If WRF-CMAQ is run in two-way coupled mode, then the EPIC model simulation uses a previously run WRF-CMAQ simulation to define the meteorological and N deposition inputs. Daily values of all soil parameters needed to compute the available soil ammonia concentrations for each of 21 agricultural production types that are either rainfed or irrigated (42 types total) are input to CMAQ. In CMAQ, the ratio of NH_4_^+^ to H^+^, typically denoted by *Γ*_*g*_, is calculated for each agriculture type and then aggregated to the model grid cell according to its fractional area. The average *Γ*_*g*_ value for the agricultural fraction combined with the nonagricultural *Γ*_*g*_, which is set to a natural background value of 20 (Zhang et al., 2010), for each grid cell is used along with ground temperature *T*_*g*_ to compute the concentration of NH_3_ gas in the soil pore space (*χ*_*g*_) in two soil layers (1 and 5 cm) according to Nemitz et al. (2000) and Pleim et al. (2013):

(1) $$\chi_{g}=F_{\text{avail }}\frac{A}{T_{g}}10^{-B}/T_{g}\Gamma_{g}$$

where *χ*_*g*_ is in μg/m^3^, $\Gamma_{g}=\frac{\left[NH_{4}^{+}\right]}{\left[H^{+}\right]}$, and A (2.7457×10^15^) and B (10,378) are constants derived from the equilibria constants, and the factor *F*_avail_ accounts for the fraction of total ammonia in the soil that is available for volatilization. The concentrations of NH_4_^+^ and H^+^ are derived from EPIC inputs for each of 42 agricultural types. The molar concentrations of H^+^ are specified by each type on an annual basis from pH values with the assumption that there is little daily variation in soil pH for managed agricultural soils. The molar concentrations of NH_4_^+^ are computed from daily EPIC estimates of the ammonia pool in the 1- and 5-cm soil layers and the soil moisture content of these layers as

(2) $${\text{NH}}_{4\;aq}^{+}=\frac{\text{L}1_{-}{\text{NH}}_{3}}{d_{1}w_{1}}$$

where L1_NH_3_ is total NH_3_ in soil layer 1, *d*_1_ is soil layer thickness of layer 1 (1 cm), and w_1_ is soil moisture. The factor *F*_avail_ in equation (1) accounts for the fraction of total soil ammonia that is in soil water solution since the total ammonia in soil includes both the ammonium in solution and sorbed on soil particles. Zhenghu and Honglang (2000) found that ammonia volatilization rates were negatively correlated with the cation exchange capacity (CEC) and clay content. Vogeler et al. (2011) found that using CEC and clay content best explained variation in the parameters for the Freundlich adsorption isotherms. Close correlation of the magnitude of NH_4_^+^ sorption to soil particles with CEC was also found by Ranjbar and Jalali (2013), Elmacı et al. (2002), Zhang et al. (2007), Zhu et al. (2011), and Yu et al. (2011). While many studies found that laboratory equilibrium experiments of ammonia soil adsorption were well explained by two or three parameter isotherm adsorption models (e.g., Sieczka & Koda, 2016; Venterea et al., 2015; Vogeler et al., 2011), they all stress that the parameters are specific to the soil condition and are therefore difficult to generalize. When we implemented the sorption isotherm model described by Venterea et al. (2015) using parameters specified by soil texture class derived from properties of agricultural soils compiled by Breuillin-Sessoms et al. (2017), the resulting available fraction was almost always less than 10% everywhere in the CONUS domain. This result does not seem to be consistent with field observations. For example, Walker et al. (2013) found an available fraction of about 55% in a corn field in eastern North Carolina (NC).

**Figure 1.**
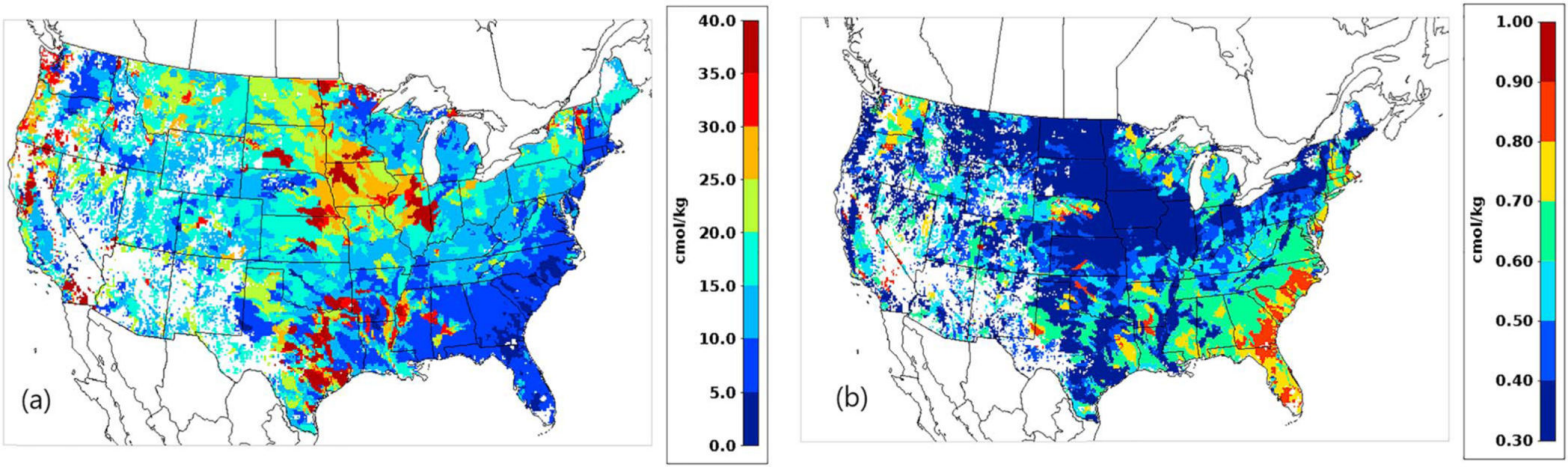
(a) The cation exchange capacity *CEC* from the EPIC model and (b) the available fraction *F*_avail_ from equation (3).

Since strong correlation of available fraction with CEC has been shown, we decided to implement a simple parameterization following the EPIC model (Williams, 1995) as

(3) $$F_{\text{avail }}=1.-0.038\times \text{CEC with }F_{\text{avail }}>0.3$$

where *F*_avail_ is used in the parameterization of volatilization in EPIC and CEC is in cmol/kg. This parameterization results in values for *F*_avail_ that range from 0.3 to 1. Figure 1 shows the CEC and *F*_avail_ values in soil layer 1 for the 12-km grid resolution CMAQ modeling domain. Clearly, this factor is a significant source of uncertainty in the bidirectional flux model that warrants more study.

#### 2.3.2. Bidirectional NH_3_ Flux Calculation

**Figure 2.**
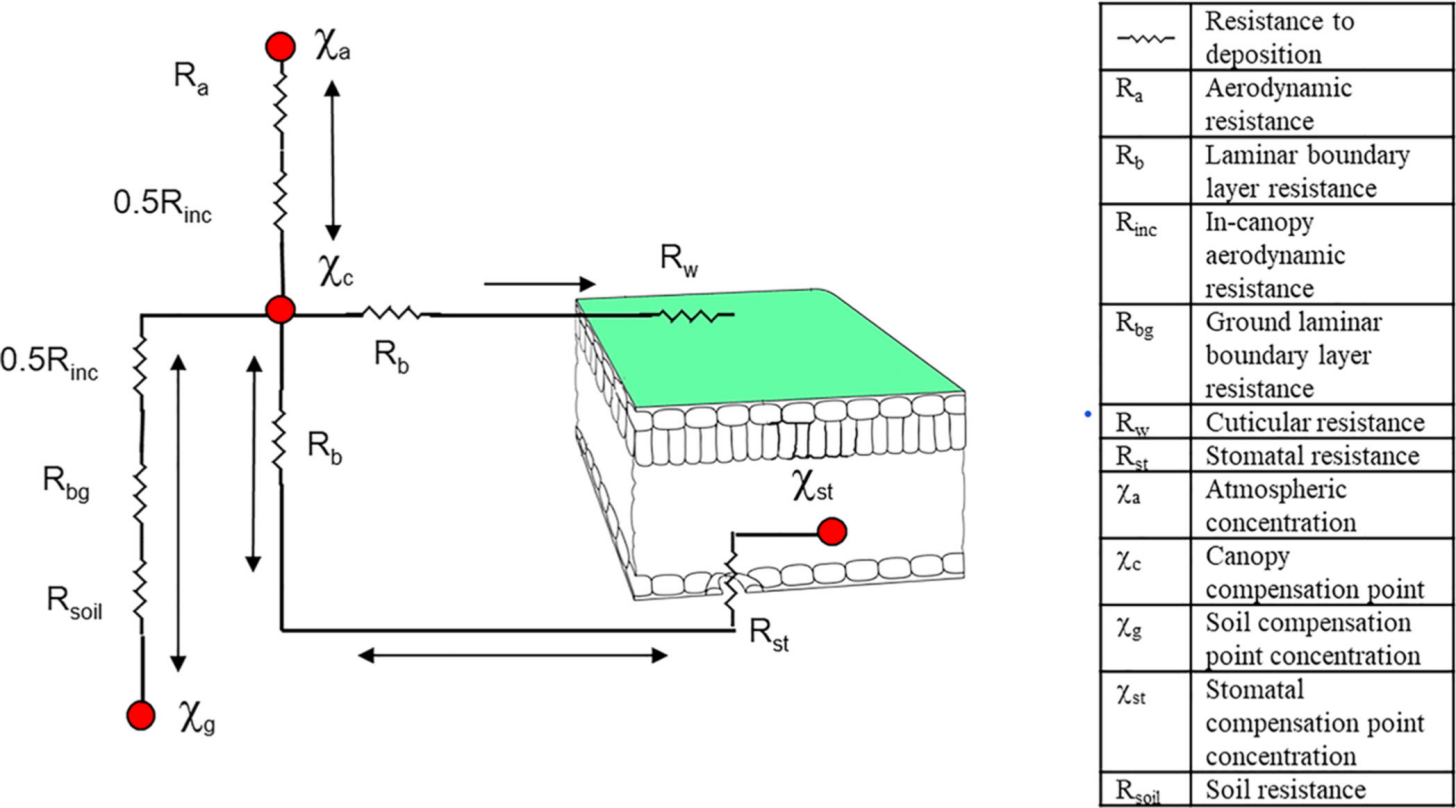
Resistance model schematic for bidirectional NH_3_ flux with leaf and soil compensation point concentrations (reproduced from Pleim et al., 2013, Figure 1)

The ammonia bidirectional flux calculation in CMAQ is a simple resistance model, which is essentially the same as presented in Pleim et al. (2013). The key difference between the bidirectional flux model and the dry deposition model is that concentrations at the surfaces are not 0. As shown in Figure 2 (reproduced from Pleim et al., 2013, Figure 1) there are compensation concentrations in the leaf stomata and the ground (soil). Thus, the fluxes via these pathways are two-way; the flux is downward (deposition) if the air concentration is greater than the surface compensation concentration and upward (emission) if the surface concentration is greater. The surface compensation concentrations at the ground and leaves are the NH_3_ concentrations in the soil pore air space or the stomatal cavity in Henry’s law equilibrium with aqueous ammonium ion and hydrogen ion in solution (in the soil water or in the apoplast leaf tissue) computed as equation (1). The soil concentrations are computed from *Γ*_*g*_ values, which are computed from EPIC inputs as described above, and stomatal concentrations are computed from *Γ*_*st*_ values specified by land use category adapted from Zhang et al. (2010). In addition to stomata and soil some models also include compensation concentration and bidirectional flux pathways for leaf litter (Nemitz et al., 2000) and leaf cuticles (Kruit et al., 2010). Field studies have often found very large values for litter emission potential, *Γ*_litter_, but also that the values are highly dynamic and poorly understood (Flechard et al., 2013). For agriculture production land the EPIC model simulates complete carbon, nitrogen, and phosphorus cycles including plant harvest and residuals. Thus, N from the leaf litter should be included in the total nitrogen pool computed by EPIC.

#### 2.3.3. Key Resistances

Complete description of the bidirectional resistance model is presented by Pleim et al. (2013). The formulations for the key resistances to leaf cuticles and soil layers are briefly summarized here. The cuticle resistance plays a particularly important role in ammonia fluxes from fertilized soils because of the large amount of NH_3_ available for volatilization from applied fertilizer and the large and rapidly growing leaf area especially during early to middle parts of the growing season (June–July). Much of the NH_3_ flux from the soil is removed by deposition to the leaves both through stomata and the leaf cuticles. Pleim et al. (2013) estimated that 78% of the ground NH_3_ emissions in a corn field in mid-July were deposited to the canopy. Estimates of 73% canopy uptake for the same field study derived from in-canopy measurements by Bash et al. (2010) agree well with the model calculations.

The cuticle resistance, *R*_*w*_, is parameterized as a function of both relative humidity (RH) and the in-canopy NH_3_ concentration following laboratory experiments described by Jones et al., 2007 and Jones et al., 2007) as

(4) $$R_{w}=\frac{1}{\mathrm{LAI}}\frac{R_{wo}}{H_{\text{eff }}}\left(\frac{\chi_{c}}{\chi_{\text{ref }}}+1\right)+(100-\max(\text{RH},60))+R_{w\text{min}}$$

where LAI is the leaf area index, *H*_eff_ is the effective Henry’s law constant that includes effects of aqueous dissociation, *χ*_*c*_ is the concentration in the canopy (μg/m^3^), RH is in percent (%), and the empirical constants are *R*_*wo*_ = 125,000 s/m, *χ*_ref_ = 1.0 μg/m^3^, and *R*_*w*min_ = 20 s/m.

Soil resistance is another key resistance for bidirectional ammonia flux because it is a critical regulator of flux from the soil where ammonia is often highly concentrated in the soil water in fertilized agriculture lands. Pleim et al. (2013) tested several formulations for soil resistance and chose the soil resistance model of Sakaguchi and Zeng (2009) based on box model comparisons to the field flux measurements described by Walker et al. (2013). Soil resistance, *R*_soil_, is parameterized as

(5) $$R_{\text{soil }}=\frac{L_{\text{dry }}}{D_{p}}$$

where *L*_dry_, the characteristic length scale accounting for tortuosity through the soil surface dry layer, is a function of soil moisture relative to saturation, and *D*_*p*_, which is the gas diffusivity through the soil pores, is computed from soil characteristics such as porosity, residual moisture content, and the slope of the retention curve. Detailed descriptions of *L*_dry_ and *D*_*p*_ are given by Pleim et al. (2013). Note that this calculation for soil resistance is also used in the PX LSM for modeling evaporation from soil (Ran et al., 2016).

## 3. Evaluation

The WRF-CMAQ-EPIC system was run for the entire year of 2016 over the CMAQ CONUS 12-km grid domain. The impacts on air quality of the bidirectional NH_3_ flux modeling system are evaluated through comparisons to ambient air concentrations from ground measurements and satellite retrievals, and wet deposition measurements. Evaluation of the FEST-C and EPIC components of the system will be presented in a forthcoming publication.

### 3.1. Model Configuration

The EPIC inputs required for the CMAQ bidirectional flux model are generated using FEST-C V1.4 with WRF-CMAQ weather and N deposition information. EPIC is configured with the variable daily curve number with depth soil water weighting for runoff estimation, modified Universal Soil Loss (MUSL) equation for water erosion, Hargreaves method for daily evapotranspiration, curve number estimate for infiltration, 4-mm slug flow method for percolation and subsurface flow computation, and Armen Kemanian method for denitrification (Doro et al., 2017; Gassman et al., 2005; Williams, 1995). The agricultural land hydrology includes tile drainage processes, and the concentration of the CO_2_ level in atmosphere is set to be 404 ppm, which represents the average background concentrations for 2016 estimated from measurements at Mauna Loa, HI (https://www.esrl.noaa.gov/gmd/ccgg/trends/). Consistent with WRF-CMAQ simulations, 2011 National Land Cover Database (NLCD; Homer et al., 2015) and corresponding county-level agricultural production area census information are used in FEST-C to generate agricultural land fractions for 42 grassland and cropland types, which are required for both EPIC and CMAQ simulations.

The WRF model version 3.8.1 was run for the CONUS domain starting on 22 December 2015 and running continuously through 31 December 2016. WRF model physics includes RRTMG shortwave and longwave radiation (Iacono et al., 2008), Pleim-Xiu land-surface model (PX-LSM; Pleim & Xiu, 1995; Xiu & Pleim, 2001), Asymmetric Convective Model version 2 planetary boundary layer (PBL) scheme (ACM2; Pleim, 2007a, 2007b), Morrison microphysics (Morrison et al., 2005), and Kain-Fritsch version 2 subgrid cloud convection model (Kain, 2004). Four-dimensional data assimilation grid nudging was used continuously for the entire simulation for temperature (T), specific humidity, and winds above PBL only, from 3-hr increments of North American Model (NAM) 12-km analyses (Gilliam & Pleim, 2010). Also, indirect soil moisture and temperature assimilation from surface analyses of 2-m T and 2-m RH were used in the PX LSM (Pleim & Gilliam, 2009; Pleim & Xiu, 2003). Lightning data assimilation using data from the National Lightning Detection Network (Orville, 2008) as described by Heath et al. (2016) helps to improve the simulation of convective precipitation. The WRF simulation was thoroughly evaluated through comparison to comprehensive surface meteorology data and compiled in a publicly accessible report (U.S. EPA, 2018)

CMAQ model version 5.3 was initialized on 22 December 2015 and run continuously through 31 December 2016. The model was configured to use the CB6 gas-phase chemical mechanism and AE7 aerosol model. Anthropogenic emissions were provided from the 2016beta Emissions Modeling Platform (U.S. EPA, 2019). The CMAQ domain is same as for WRF but reduced by five grid cells on each of the four boundaries. Initial and boundary conditions were extracted from hemispheric WRF-CMAQ simulations, which used a similar but slightly different configuration for CMAQ. CMAQ was run both with and without the new bidirectional NH_3_ flux model. The run with the bidirectional model is referred to as the *bidi* run, while the run without the bidirectional model is referred to as *base*. The base simulation uses agricultural fertilizer emissions using monthly county level emission factors based on early versions of FEST-C and CMAQ with the bidirectional ammonia flux model as described in U.S. EPA (2019).

### 3.2. Comparisons to Observations

When ammonia gas is released into the air some will transform into aerosol form, while the rest remains in the gas phase. The amount that becomes aerosol ammonium (NH_4_^+^) depends on the concentrations of anions in the air, typically sulfate, nitrate, and chloride, which can combine with ammonium to form ammonium sulfate ((NH_4_)_2_SO_4_), ammonium bisulfate (NH_4_HSO_4_), ammonium nitrate (NH_4_NO_3_), and ammonium chloride (NH_4_Cl). Ideally, an evaluation of ammonia fluxes would include comparisons of model predictions to ambient concentration of NHx that would include both gas and aerosol forms (NHx = NH_3_ + NH_4_^+^). Unfortunately, NHx is not currently measured at national networks in the U.S.. Therefore, evaluation of CMAQ entails a combination of gas, aerosol, and wet deposition measurements. The CMAQ model with the bidirectional NH_3_ surface flux is evaluated for surface level NH_3_ concentration through comparison to surface site measurements at the National Atmospheric Deposition Program (NADP) Ammonia Monitoring Network (AMoN) and surface NH_3_ concentration retrievals from the Cross-Track Infrared Sounder (CrIS) satellite sensor. Wet deposition of NH_4_^+^ is evaluated through comparison to the NADP National Trends Network (NTN). Ammonium and sulfate aerosol components are evaluated through comparisons to the Chemical Speciation Network (CSN), the Clean Air Status and Trends Network (CASTNET), and Southeastern Aerosol Research and Characterization (SEARCH; Edgerton et al., 2005) networks.

### 3.3. Ammonia Gas Evaluation With AMON

**Figure 3.**
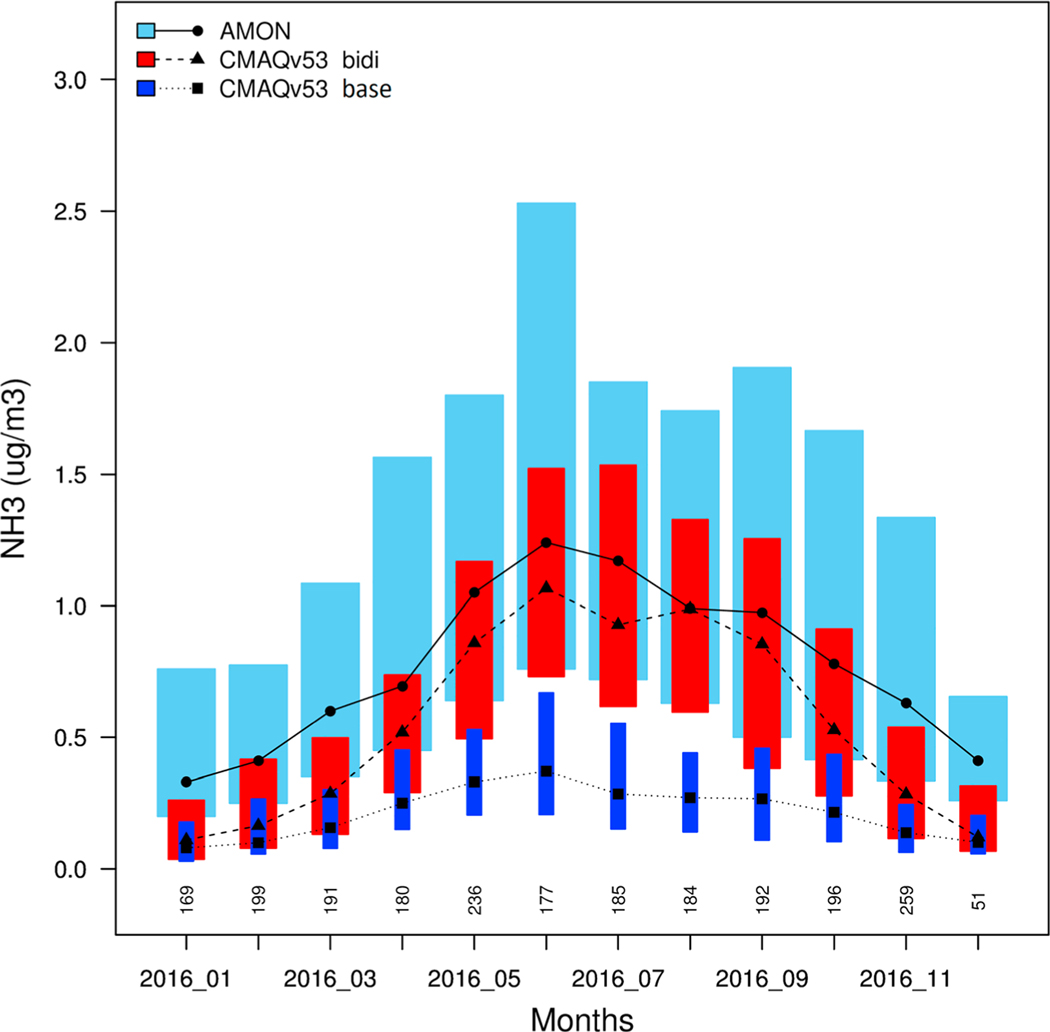
Monthly average NH_3_ concentrations at Ammonia Monitoring Network (AMoN) sites for bidi (red) and base (dark blue) model runs and measurements from AMoN (light blue). The black symbols with connecting lines indicate median values. Lower and upper ends of the boxes indicate the 25th and 75th percentiles of each distribution. The number of observations per month is shown above the *x* axis.

The AMoN measures ambient gas-phase ammonia on a 2-week accumulated average basis at 66 sites across the CONUS (Puchalski et al., 2011). Only the data flagged as valid (AMoN valid flag “A” or “B” and replicate not equal to “T”) are included in the analysis. Figure 3 shows monthly averages of all valid AMoN measurements paired with model results extracted at AMoN site locations and averaged over the same 2-week periods and then monthly. The two sets of CMAQ model results compare model simulations with and without the bidirectional treatment of ammonia fluxes using otherwise identical model configurations and inputs. The base simulation (labeled *base* in subsequent figures and discussion) greatly underpredicts NH_3_ concentrations compared to AMoN, particularly for the warm season (e.g., May–September), when the monthly median model values are biased low by about 75%. The simulation with the bidirectional fluxes (labeled *bidi* in subsequent figures and discussion) predicts much greater NH_3_ concentrations that compare more favorably with the AMoN measurements particularly during the growing season. The median values from the bidi run are within 20% of the observed median values for the warm season (May–September), although the modeled interquartile ranges (length of bars) are smaller than the observations. For winter months, December through March, the bidi model underpredicts at the AMoN sites by an average of 62%. However, since NH_3_ volatilization is an exponential function of temperature (equation (1)), NH_3_ from managed agriculture soils is likely to be a much smaller fraction of total ammonia emissions in the winter than during the growing season, which suggests that other sources of ammonia emission are underestimated by the model.

**Figure 4.**
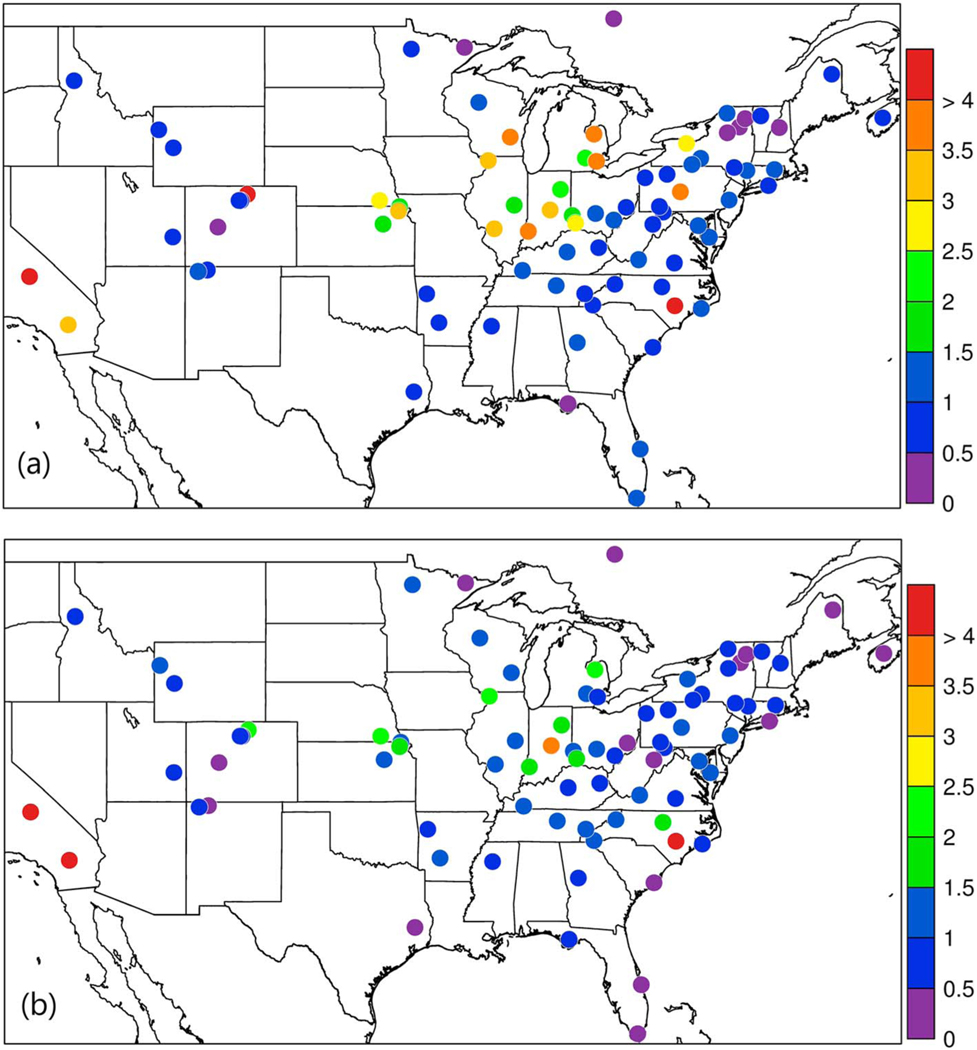
Ammonia concentrations (μg/m^3^) at Ammonia Monitoring Network (AMoN) sites averaged for May–September 2016 for (a) AMoN measurements and (b) Community Multiscale Air Quality (CMAQ) bidi.

Measured ammonia concentrations averaged over the warm season (May–September) at each AMoN site where at least 75% of the data were valid, and comparable modeled (bidi) concentrations are shown in Figure 4. Unfortunately, much of the most intensely cultivated lands, particularly the Great Plains from Northern Texas up through the Dakotas and Minnesota, are not well covered by the AMoN network. The model and measurements are in general agreement that the higher NH_3_ concentrations occur in the Central Valley of CA (only one site), the Central Plains as represented by only four sites clustered in southeast Nebraska and northeast Kansas, and the Corn Belt of Illinois, Indiana, Ohio, Wisconsin, and Michigan. Note that the high concentrations in southeastern NC shown in the measurements and model are in an area of concentrated hog facilities.

**Figure 5.**
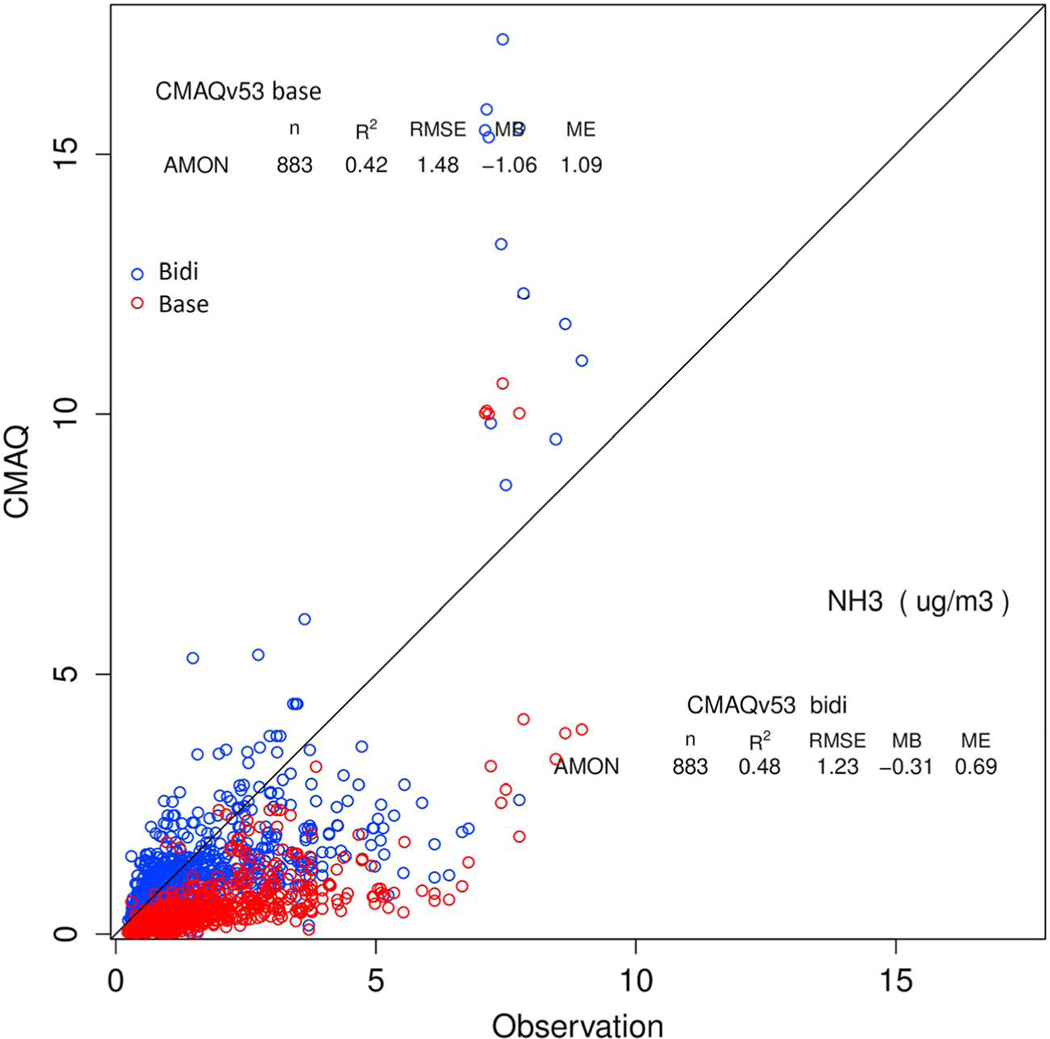
Observed vs modeled NH_3_ concentration at Ammonia Monitoring Network (AMoN) sites over May to September 2016 for bidi (blue) and base (red).

The scatterplot of measured and modeled concentrations at AMoN sites for May through September (Figure 5) shows considerable scatter for both model simulations but greatly reduced bias, slightly reduced error, and increased correlation for the bidi case compared to the base case.

### 3.4. Evaluation of Aerosols

The partitioning of total ammonia (NHx) into gas-phase and aerosol-phase ammonia depends on concentrations of the gas-phase NH_3_ and HNO_3_ and aerosol-phase ammonium, nitrate, sulfate, calcium, potassium, magnesium, sodium, and chloride. The thermodynamic equilibrium among these species, which depends strongly on temperature and humidity, is computed by the ISORROPIA v2.1 (Fountoukis & Nenes, 2007; Nenes et al., 1998). Note that sulfate exists entirely in the aerosol phase and depends on SO_2_ emissions and both gas and aqueous phase oxidation of SO_2_ to SO_4_^2-^. Modeled concentrations of gas phase NH_3_ and aerosol phase NH_4_^+^ are influenced by many other chemical, physical, and meteorological processes in addition to ammonia emissions and deposition. Thus, evaluation of NH_3_ emission models should also include evaluation of aerosol species such as ammonium, nitrate, and sulfate.

**Figure 6.**
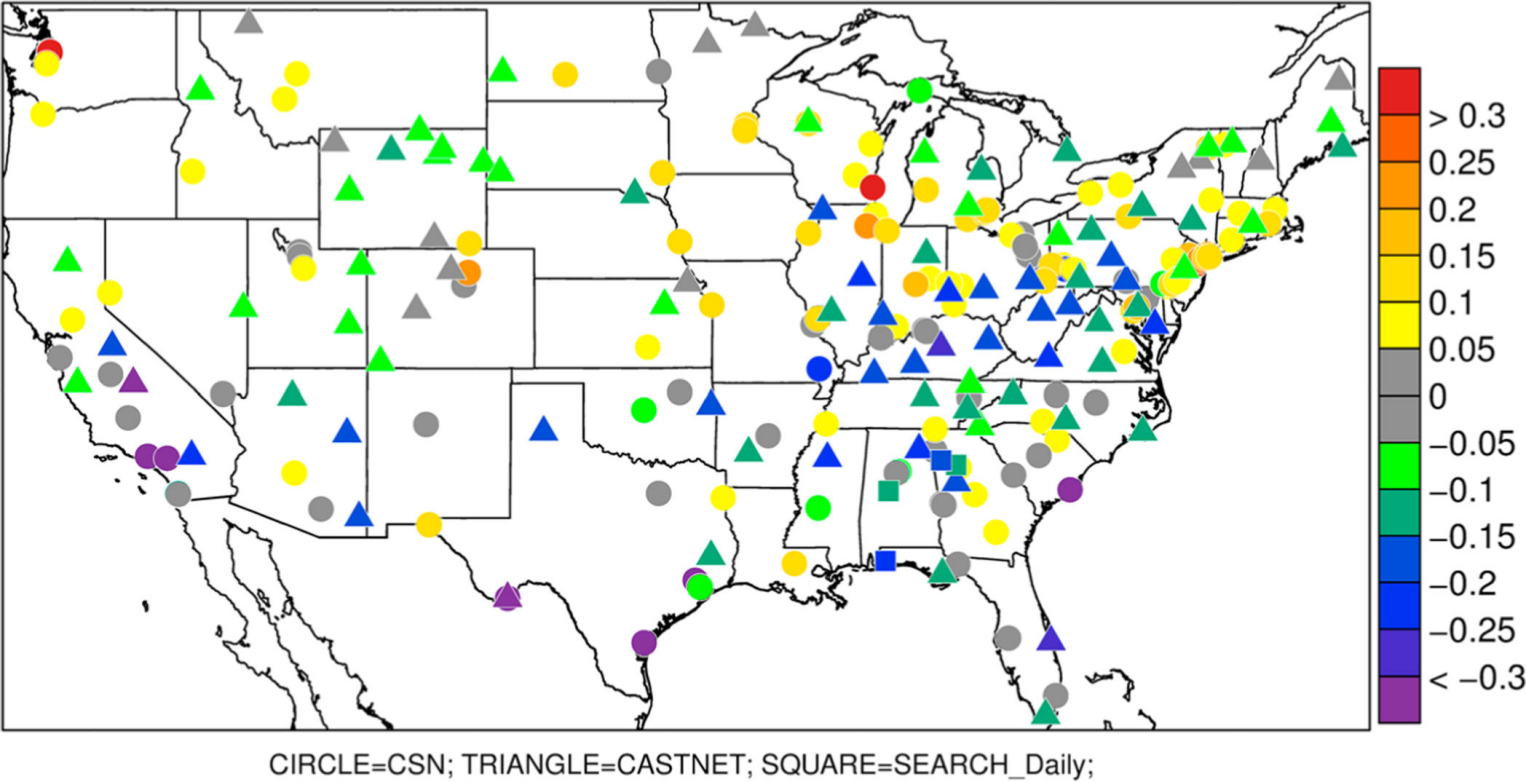
Biases in modeled ammonium PM_2.5_ (μg/m^3^) compared to Chemical Speciation Network (CSN; circles), CASTNet (triangles), and Southeastern Aerosol Research and Characterization (SEARCH; squares) networks averaged over May to September 2016.

There are two national networks that measure NH_4_^+^ aerosol components, CSN and CASTNET, in addition to the SEARCH regional network. CSN and SEARCH measure speciated aerosols from 24-hr average filter samples every 3 days and CASTNET uses weekly filter pack measurements. Figure 6 shows the bidi model bias of aerosol PM_2.5_ NH_4_^+^ concentrations averaged over the warm season (May–September). At most CSN sites (other than CA) the model overestimates the reported measured concentrations while the model generally underestimates concentrations at CASTNET and SEARCH sites. At locations where CSN sites are close to CASTNET and SEARCH sites the model bias is often of opposite sign. Part of this discrepancy may be that CSN is designed to represent urban sites, while CASTNET is designed to characterize background rural locations. However, discrepancies between CSN and SEARCH may be more indicative of measurement artifacts. Pye et al. (2018) analyzed ammonium to sulfate ratios measured by different instruments and compared to thermodynamic models during the Southern Oxidant and Aerosol Study (SOAS). They concluded that CSN tends to underestimate NH_4_^+^ concentrations, while SEARCH measurements agree well with the thermodynamic models and the SOAS Monitor for AeRosols and Gases in Ambient air (MARGA) measurements. Although Pye et al. (2018) did not analyze the NH_4_^+^ measurements from CASTNet, Figure 6 shows that CASTNet is similar to SEARCH when sites are in close proximity. Therefore, CASTNet measurements of NH_4_^+^ are likely more realistic than CSN. Considering the likely greater fidelity of CASTNet and SEARCH than CSN, Figure 6 suggests that CMAQ tends to underestimate NH_4_^+^ (e.g., biases at CASTNet eastern sites are mostly −0.1 to −0.25 μg/m^3^ and at western sites mostly −0.05 to −0.2 μg/m^3^).

**Figure 7.**
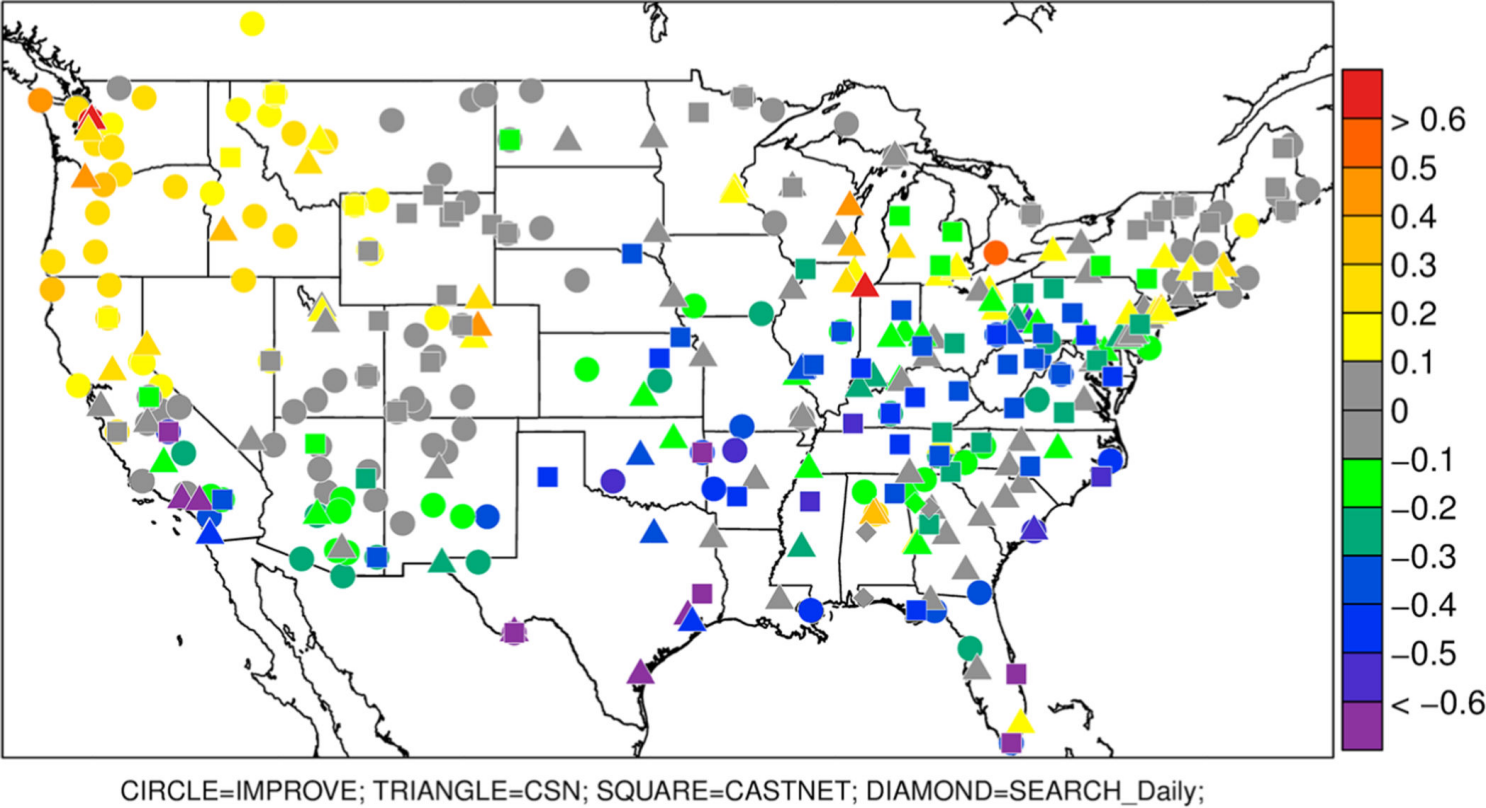
Biases in modeled sulfate PM_2.5_ (μg/m^3^) compared to Interagency Monitoring of PROtected Environments (IMPROVE; circles), Chemical Speciation Network (CSN; triangles), CASTNET (squares), and Southeastern Aerosol Research and Characterization (SEARCH; diamonds) networks averaged over May to September 2016.

Given the strong dependence of NH_4_^+^ on SO_4_^2−^, comparison to ambient sulfate measurements is also relevant to ammonia model evaluation. Figure 7 shows model PM_2.5_ SO_4_^2−^ concentrations averaged over the warm season (May–September) compared to measured SO_4_^2−^ concentrations from the Interagency Monitoring of PROtected Environments (IMPROVE), CSN, CASTNet, and SEARCH networks. With the exceptions of the northwest and a few sites around the Great Lakes and New England, the model generally underestimates SO_4_^2−^ compared to all four networks. These results suggest that the model’s tendency to underestimate NH_4_^+^ may be in part related to underestimation of SO_4_^2−^. Modeled and measured PM_2.5_ NH_4_^+^ and PM_2.5_ SO_4_^2−^ concentrations have very similar spatial patterns (not shown) with peak concentrations along the Ohio River where SO_2_ emissions are greatest.

### 3.5. Evaluation of Wet Deposition of Ammonium and Nitrate

**Figure 8.**
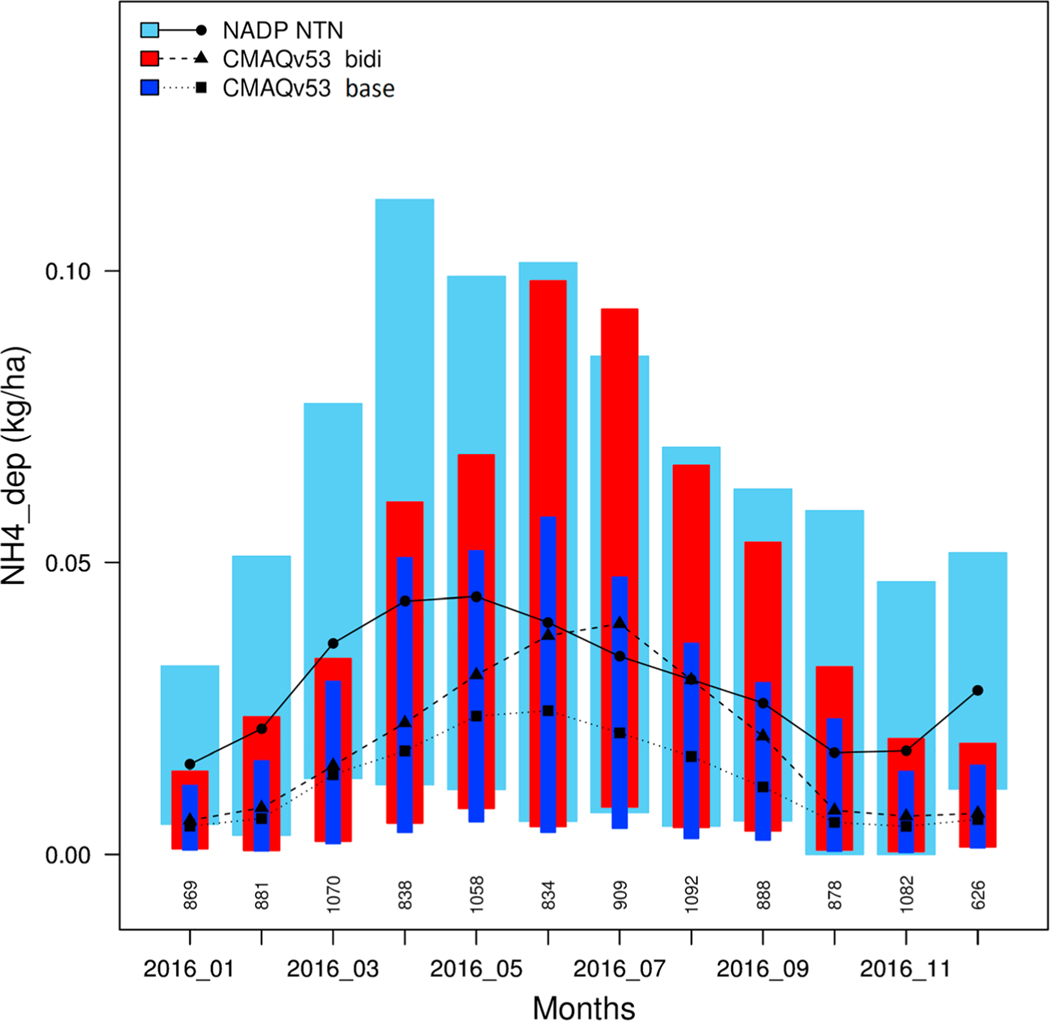
Monthly average ammonium wet deposition at National Atmospheric Deposition Program (NADP) National Trends Network (NTN) sites from measurements (light blue), Community Multiscale Air Quality (CMAQ) bidi (red) and CMAQ base (dark blue). The black symbols with connecting lines indicate median values. Lower and upper ends of the boxes indicate the 25th and 75th percentiles of each distribution.

**Figure 9.**
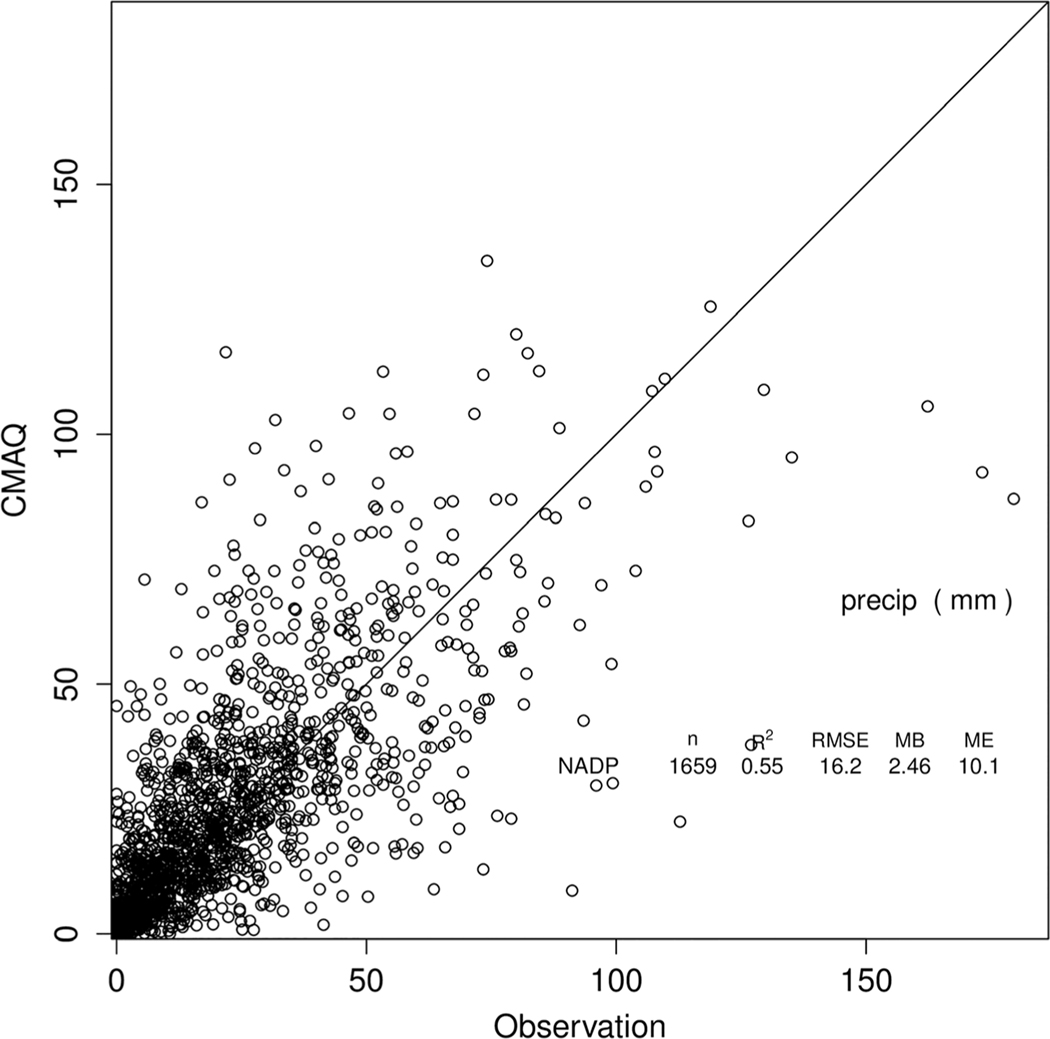
Comparison of modeled weekly precipitation for April and May 2016 to measurements at National Trends Network (NTN) sites.

The NADP NTN wet deposition network measures precipitation chemistry on an accumulated weekly basis. Since precipitation amount is also measured, chemical wet deposition flux is determined. An advantage of wet deposition measurements is that both gas and aerosol phase NHx should be nearly equally scavenged by precipitation since ammonia gas will be almost entirely dissolved in cloud water (Seinfeld & Pandis, 1998), which means that these comparisons are less affected by gas-aerosol partitioning. Figure 8 shows monthly distributions of average NH_4_^+^ wet deposition in kg/ha per week from the NTN measurements compared to CMAQ. During the summer months (June–September), the CMAQ bidi run agrees well with NTN for both the median values and the 25–75% distributions, while the base is much lower. Observed seasonality, however, is not well replicated by either model simulation. The NTN data show peak wet NH_4_^+^ deposition in April and May, while CMAQ results peak in June and July. A possible reason for under prediction of wet deposition in the springtime months could be underprediction of the precipitation. However, Figure 9 shows that weekly accumulated precipitation at the NTN sites is not underpredicted by the model for April and May. Note that Bash et al. (2013) also found high wet deposition amounts in April and May of 2011 that the models, both base and bidirectional, underpredicted.

**Figure 10.**
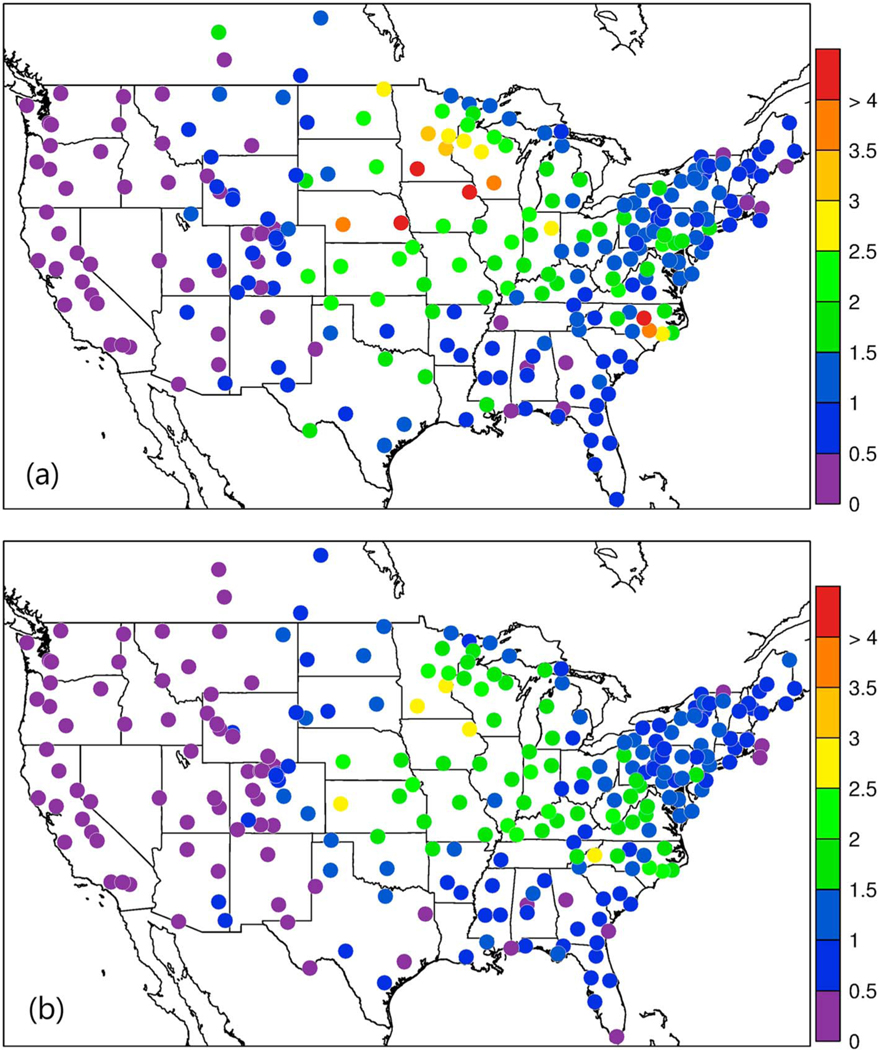
Ammonium wet deposition at National Atmospheric Deposition Program (NADP) National Trends Network (NTN) sites accumulated for May–September 2016 for (a) NADP NTN measurements and (b) Community Multiscale Air Quality (CMAQ) in kg/ha.

Spatial plots of NADP NTN measured and CMAQ bidi modeled NH_4_^+^ wet deposition for the warm season (May–September) are shown in Figure 10. While the model reproduces well the overall pattern of high and low values, the model shows large negative biases at sites mainly in the northern Plains and eastward into Minnesota, Iowa, and Wisconsin. It is interesting that the greatest observed values are near the concentrated hog production operations around Iowa and southeastern NC, while the model does not show such peak values in these regions. This suggests that either NH_3_ emissions from hog operations are underestimated or the wet scavenging of gas and/or aerosol ammonia is underestimated.

### 3.6. Cross-Track Infrared Sounder Satellite

**Figure 11.**
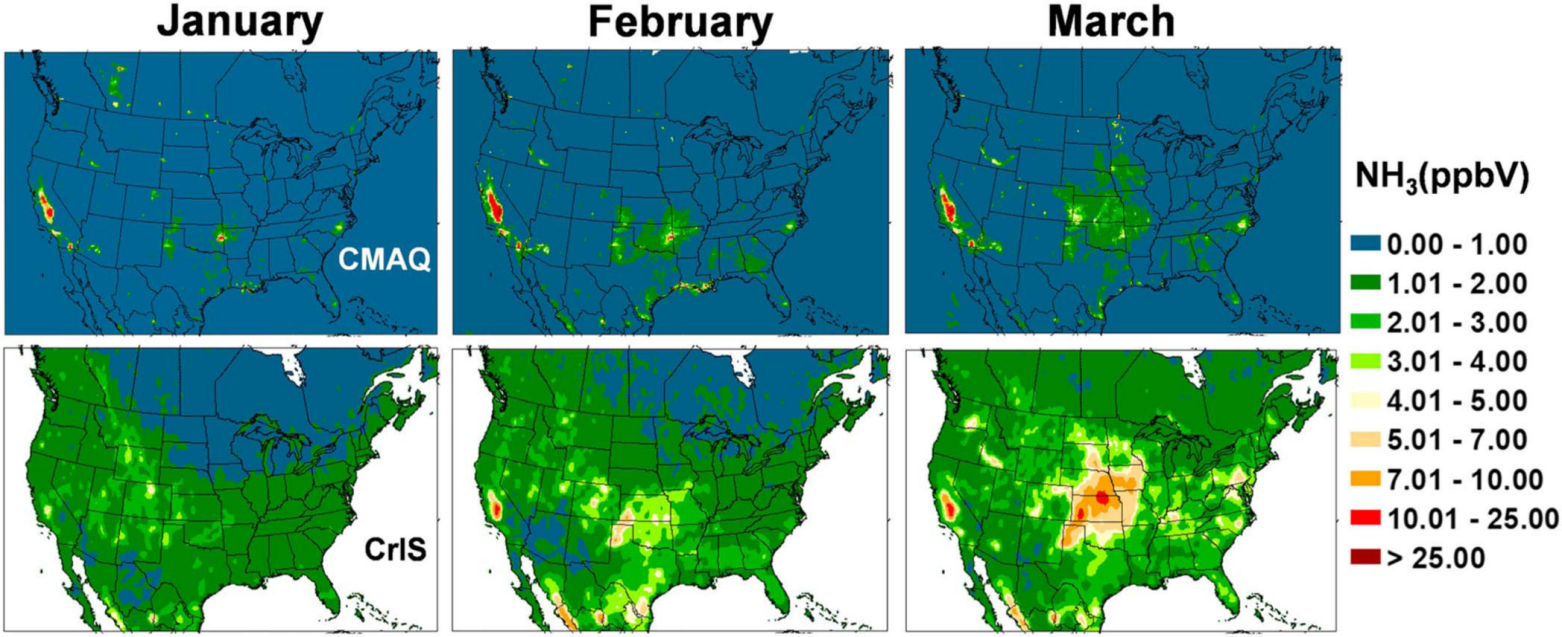
Comparison of ground level NH_3_ concentrations between Community Multiscale Air Quality (CMAQ) bidi (upper maps), and Cross-Track Infrared Sounder (CrIS) satellite retrievals (lower maps), averaged for January, February, and March 2016.

**Figure 12.**
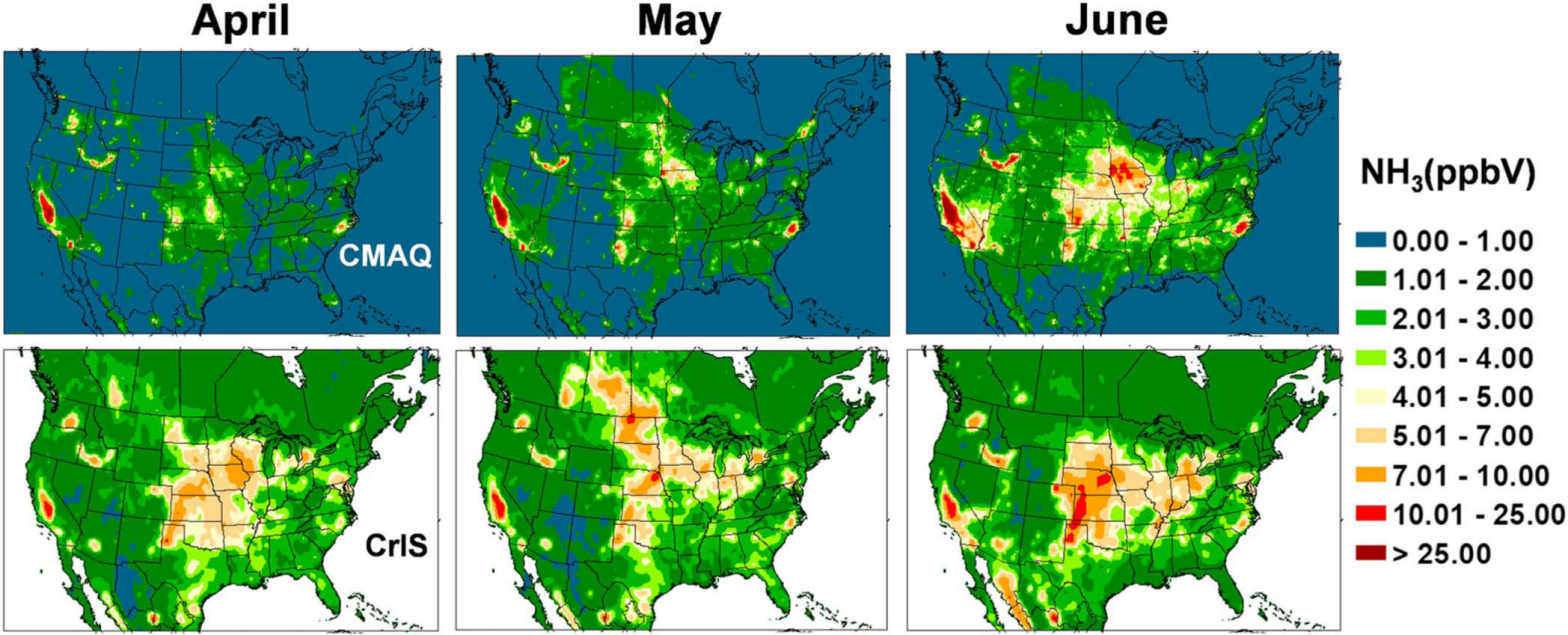
Comparison of ground level NH_3_ concentrations between Community Multiscale Air Quality (CMAQ) bidi (upper maps), and Cross-Track Infrared Sounder (CrIS) satellite retrievals (lower maps), averaged for April, May, and June 2016.

**Figure 13.**
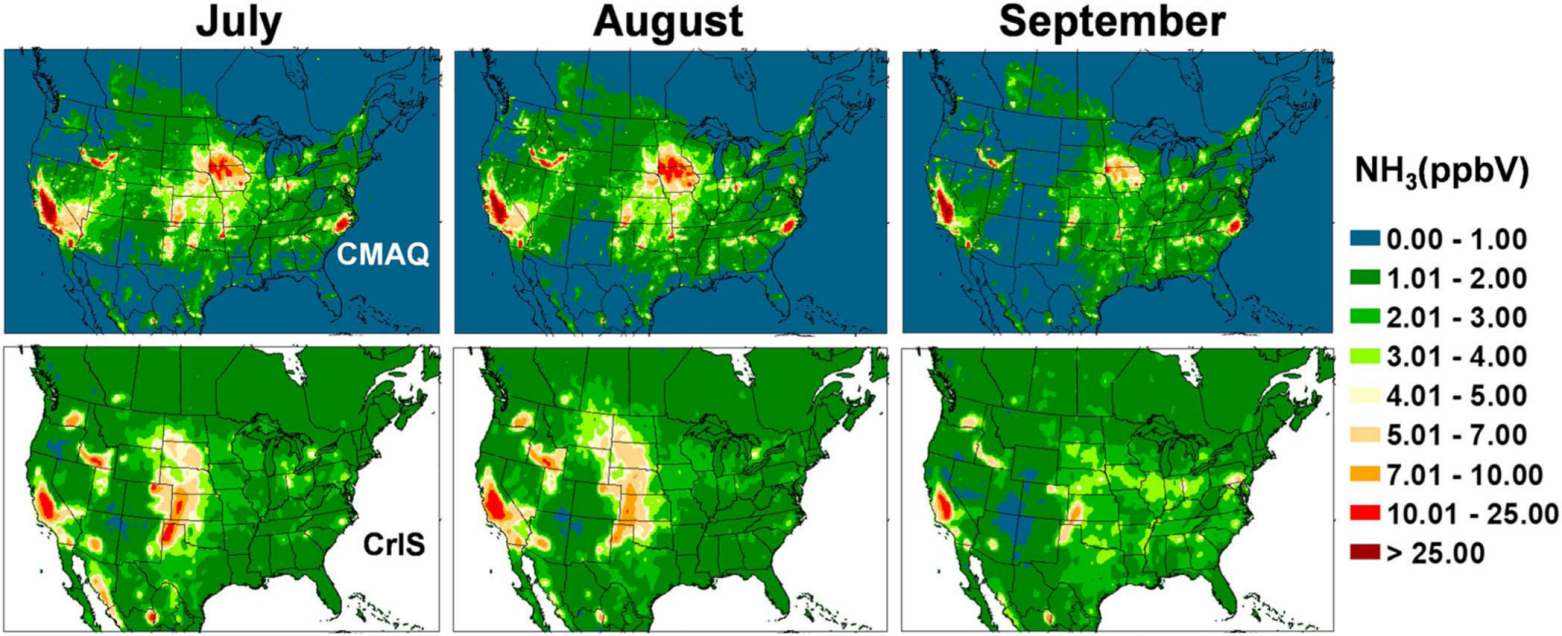
Comparison of ground level NH_3_ concentrations between Community Multiscale Air Quality (CMAQ) bidi (upper maps), and Cross-Track Infrared Sounder (CrIS) satellite retrievals (lower maps), averaged for July, August, and September 2016.

**Figure 14.**
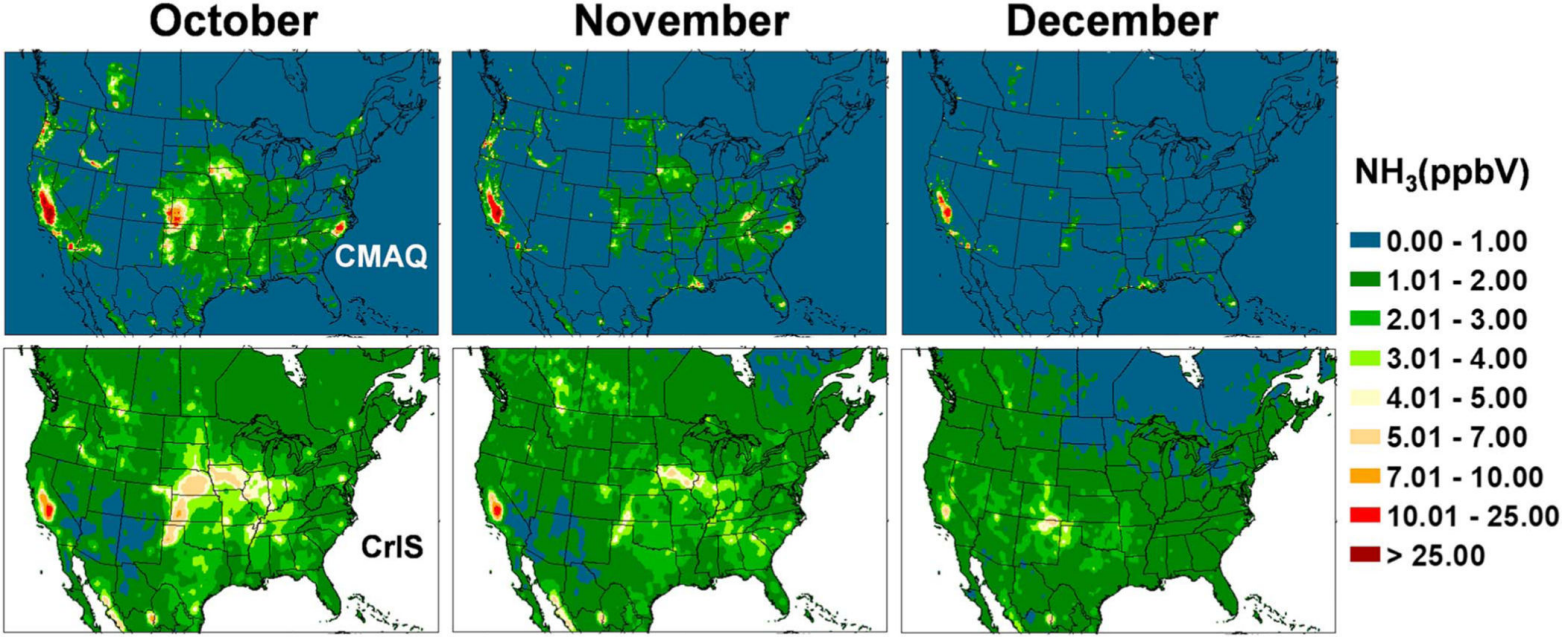
Comparison of ground level NH_3_ concentrations between Community Multiscale Air Quality (CMAQ) bidi (upper maps), and Cross-Track Infrared Sounder (CrIS) satellite retrievals (lower maps), averaged for October, November, and December 2016.

The Cross-Track Infrared Sounder Satellite (CrIS) instrument is deployed aboard polar orbiting Suomi NPOESS Preparatory Project (SNPP) satellite in a Sun-synchronous orbit with overpass times of ~01:30 and ~13:30 local time. NH_3_ is obtained from CrIS radiance measurements by applying the CrIS Fast Physical Retrieval (CFPR) algorithm, which provides ammonia volume mixing ratio profiles; the peak sensitivity of the CFPR retrieval is in the boundary layer, typically around 850–750 hPa, but it can be close to the surface under ideal conditions (Shephard & Cady-Pereira, 2015). CrIS NH_3_ has been evaluated against measurements from Fourier Transform Infrared Radiometer (FTIR) instruments from the Network for the Detection of Atmospheric Composition Change (NDACC; Dammers et al., 2017); CrIS NH_3_ showed good agreement with the FTIR, though biased slightly low for low amounts. Level 3 surface volume mixing ratio monthly average fields at 0.1-degree resolution derived from daytime (13:30 LT) retrievals are re-gridded to the CMAQ 12-km grid cells. The CrIS values are presented in Figures 11–14 along with similar maps of CMAQ bidi NH_3_ concentrations averaged over each month from daily 13:30 values. Note that there is not an exact correspondence between observed and modeled monthly averages because CrIS, due to clouds, does not return valid data every day of the month, although there are usually 20–30 valid observations per grid cell.

**Figure 15.**
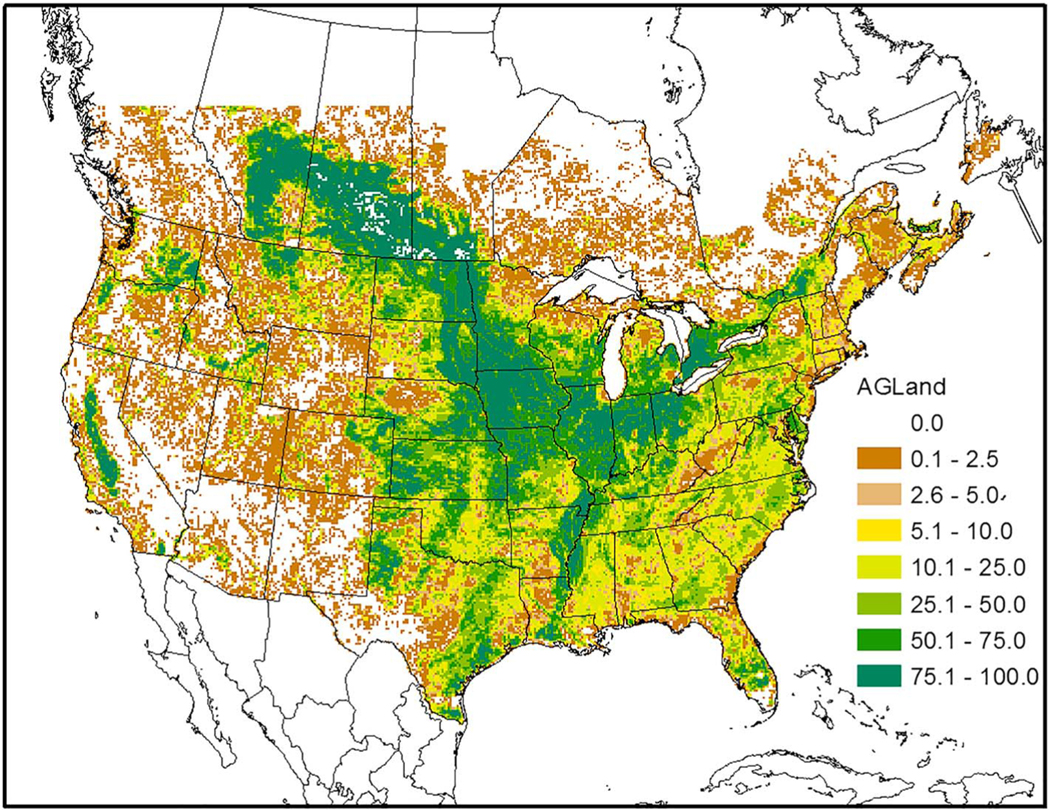
FEST-C generated area fraction of each 12 km grid cell classified as cultivated crops and pasture/hay from National Land Cover Database (NLCD) in the U.S. and Moderate Resolution Imaging Spectroradiometer (MODIS) land use data for Canada and Mexico.

The CrIS monthly average concentration maps reveal far more information on the spatial distribution of ammonia concentrations than can be discerned from the AMoN network. An obvious difference between the CrIS maps and the model maps is in very low concentration conditions, especially in the colder months, where the background color is different. Since the detection limit for the CrIS retrievals is about ~0.5 to 1 ppb (Kharol et al., 2018; Shephard & Cady-Pereira, 2015), and nondetects have not been accounted for in this Level 3 product, the lowest gridded values are around 1 ppb while the model does not have a lower limit so remote areas have values very close to zero. Over high emission land areas the model results are much more similar to the CrIS results. Comparing both the CrIS and model plots to the agriculture area fraction in each grid cell (Figure 15) shows that the highest NH_3_ concentrations do not correspond to the areas with greatest crop and pasture coverage, which indicates that simple schemes that scale emissions by crop area or specify *Γ*_*g*_ values by land-use categories, are not adequate. Factors such as fertilizer amount and type, soil type, soil moisture, and soil pH are all critical factors that influence NH_3_ flux.

Monthly average 13:30 NH_3_ concentrations from the bidi model compared to the CrIS data show general similarity of spatial distributions with highest concentrations in June and July over the United States. Compared to CrIS, the model underpredicts NH_3_ concentrations in the spring (March–May) over most of the modeled region but overpredicts in the CA Central Valley all year round. Another significant difference is that modeled NH_3_ is much greater than CrIS NH_3_ in Iowa and southeastern NC, which are both hot spots for intensive hog production. This is an opposite result from the NH_4_^+^ wet deposition comparisons at NADP NTN sites in these areas, where the model underestimates NH_4_^+^ wet deposition (Figure 10). Thus, these results support the hypothesis that scavenging of NHx is underestimated rather than an underestimation of emissions from hog facilities. Since the model substantially overestimates gas NH_3_ concentrations compared to CrIS but does not seem to overestimate aerosol NH_4_^+^ concentrations (Figure 6; although there are not many measurements sites in these areas other than the biased CSN sites), this suggests that scavenging of NH_3_ gas in particular may be underestimated by the model.

**Figure 16.**
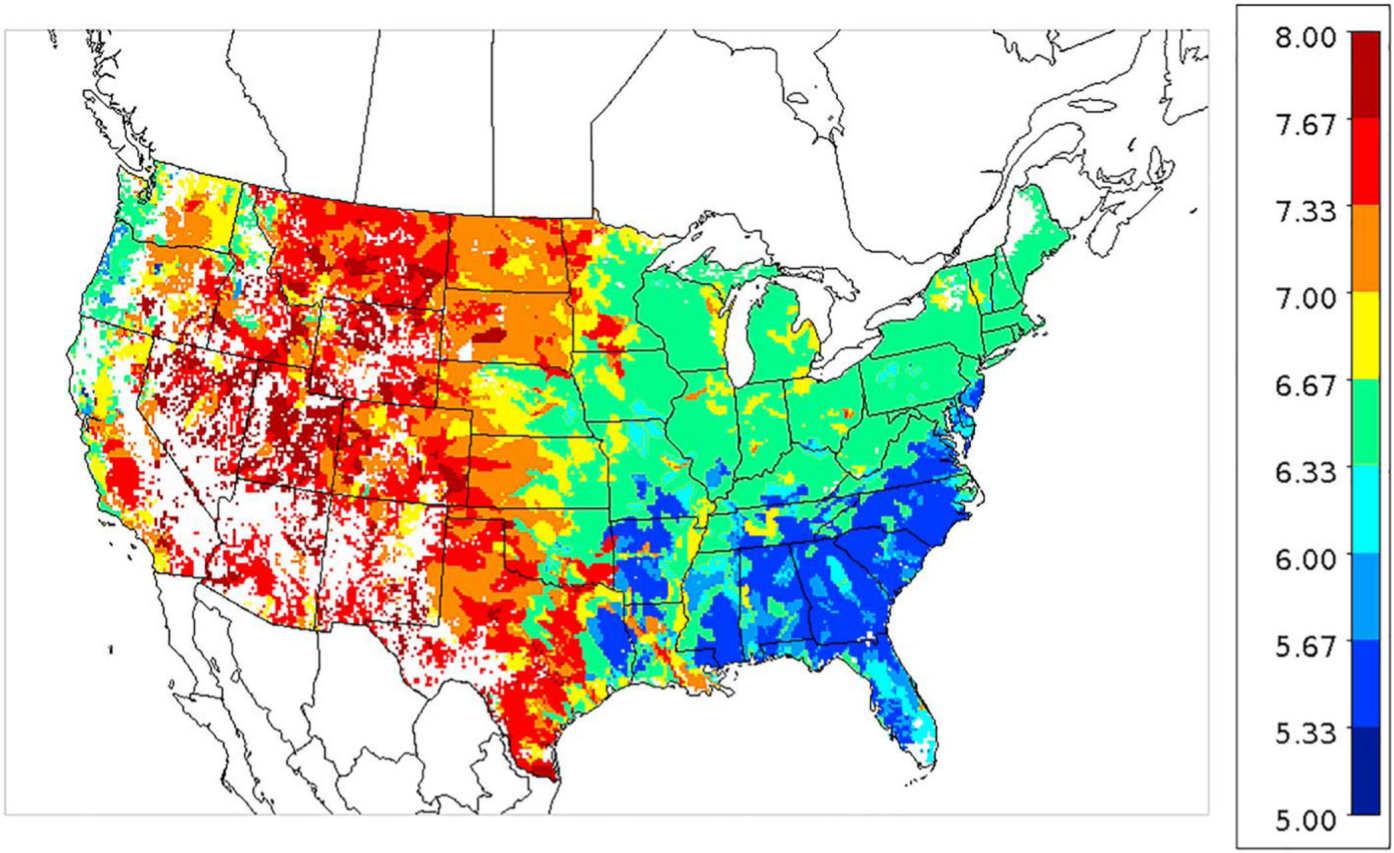
Soil pH averaged over all 42 crop types from Environmental Policy Integrated Climate (EPIC) model simulation for 2016.

A striking feature of both model and CrIS maps is the high NH_3_ concentrations in the north Texas panhandle across the Oklahoma panhandle, up through western Kansas and into Nebraska even though the fractional cropland coverage is less than many other areas with lower concentrations. Concentrations in these areas are very high in the CrIS maps from March through August, while the model shows comparable concentrations mainly in June through August. One possible reason for the relatively high NH_3_ concentrations in the western High Plains is the higher soil pH values, as is shown in Figure 16 and discussed further in the next section. Also, the lower NH_3_ values in the agricultural areas further east, near the Ohio River, may be due to more NHx in the aerosol phase because of high SO_2_ emissions and high aerosol sulfate concentrations.

## 4. Discussion

Overall the bidi model seems to underpredict ammonia gas and ammonium aerosol concentrations but to a far lesser degree than the base model. For example, the model shows a low bias in NH_3_ concentration of 20% compared to AMoN measurements and a low bias in NH_4_^+^ aerosol concentration of 35% compared to CASTNET measurements averaged over the warm season (May–September). This suggests that net surface fluxes from agricultural soils plus other NH_3_ emissions may be underestimated. Comparison of monthly averaged concentrations to CrIS retrievals shows that during the warmer months, the modeled NH_3_ concentrations are less than CrIS in most of the predominantly agricultural regions. However, a few areas stand out where the model concentrations are consistently greater than CrIS over many months. Two areas overpredicted compared to CrIS are the northern half of Iowa into southern Minnesota and southeastern NC, which are the two biggest hog producing areas in the country. The Central Valley of CA is also persistently overpredicted compared to CrIS. Since peak sensitivity in the Central Valley for CrIS retrievals is at about 900–800 hPa, and the PBL mixed layer is often quite shallow, especially in the cooler months, uncertainty at the surface is much greater with substantial high and low biases. The Central Valley includes intensive agricultural production, much of which is irrigated, and other significant sources of ammonia emissions such as animal feeding operations and mobile sources. While analysis of other (nonfertilizer) NH_3_ emission sources such as from animal feeding operations is beyond the scope of this paper, it is interesting to investigate episodes of high NH_3_ concentration associated with very large upward surface fluxes from the NH_3_ bidirectional model.

**Figure 17.**
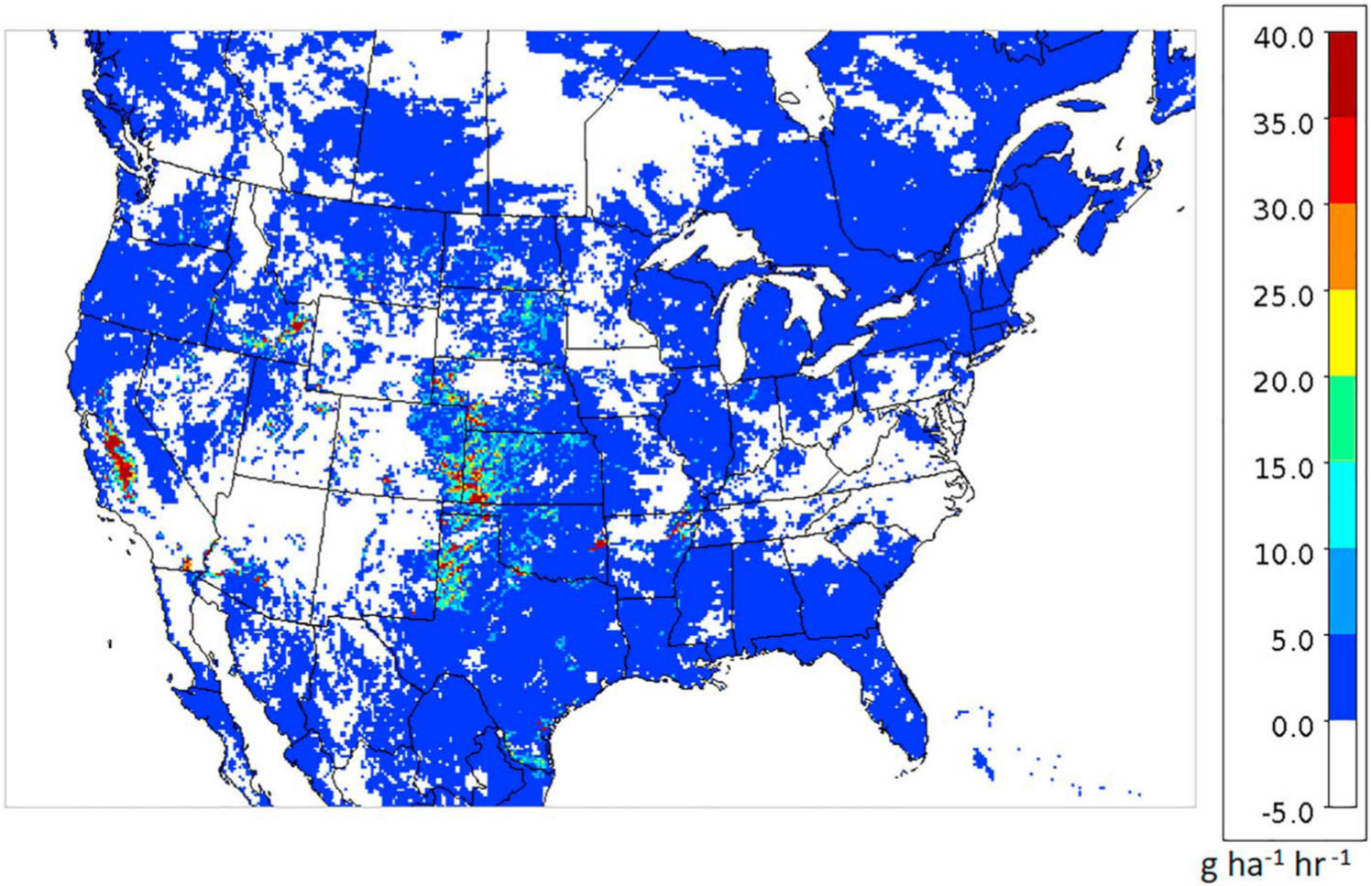
Ammonia surface flux (g · ha^−1^ · hr^−1^) on 24 June 2016 at 20 UTC.

Figure 17 shows an example of the surface flux of NH_3_ on June 24 at 20 UTC where positive values indicate upward evasive fluxes and negative values are downward dry deposition fluxes. The white areas indicate either nearly zero flux (e.g., ocean) or negative (deposition) fluxes. In the colored areas the flux is upward from the surface to the air. While the areas with the greatest upward flux varies day to day depending on meteorology, soil chemistry, crop type, and growth stage, the same areas in the Central Valley of CA, the Snake River Valley in Idaho, and the western High Plains in northern Texas, western Kansas, Eastern Colorado, and South Dakota often stand out with high upward fluxes. The relatively high soil pH in these areas as shown in Figure 16 is a key reason for the large fluxes. The fluxes are governed by the concentration gradient between surface and air, and the soil concentrations are proportional to *Γ*_*g*_ as shown in Equation (1). Since *Γ*_*g*_ is the ratio of ammonium ion to the hydronium ion, one unit difference in pH translates to a factor of 10 difference in *Γ*_*g*_.

To better understand the very high concentrations in the Central Valley of CA simulated by the CMAQ bidi compared to CrIS (Figures 11–14), we investigate the model calculations for selected extreme high concentration episodes. For example, the peak modeled NH_3_ concentration on 30 June of 233 ppb occurred at 03 UTC (8 p.m. PDT) in the San Joaquin Valley about halfway between Fresno and Bakersfield. As is typical of most areas in the Central Valley, this particular 12- × 12-km grid cell is mostly agriculture with 65% coverage by crops and 15% coverage by fertilized grasslands. Although there are four dominant crop types and four main grass types, the high value of *Γ*_*g*_ is mainly the result of irrigated cotton even though it only accounts for 8.8% areal coverage. The reason for the dominance of irrigated cotton in this cell is a combination of high pH (7.75), relatively dry soil (0.08 by volume) and very high amount of ammonia loading in the surface soil layer (29.6 kg/ha). These values result in a calculation for *Γ*_*g*_ of 15,000,000, which, when weighted by the 8.8% area, contributes 1,300,000 to the grid cell average of 1.9 million. The ammonia loading in this instance is anomalous compared to the other main crop types in this cell which range from 0.26 kg/ha for irrigated other crops to 2.8 for irrigated alfalfa. Other areas with significant coverage by irrigated cotton such as in the Mississippi River Valley do not result in such high *Γ*_*g*_ values and fluxes even though there are occasionally similar large amounts of soil ammonia because of lower pH and wetter soil conditions.

In another location in the Central Valley near Merced on 1 June at 03 UTC (8p.m. PDT) the model simulated NH_3_ concentration of 214 ppb. While the largest crop fraction in this cell is classified as irrigated other crops (38%), which is mostly almond groves, the NH_3_ fluxes are dominated by extremely high *Γ*_*g*_ values for irrigated corn silage, which only accounts for 12% of the area. Again, the main reason for the high calculation of *Γ*_*g*_ is the anomalously high soil ammonia loading from FEST-C EPIC (28.3 kg/ha). Thus, it is not a single crop type responsible for the episodes of very high fluxes that result in extreme NH_3_ concentrations in the Central Valley but rather sporadic unrealistically high soil ammonia loading for different crop types. The application of the EPIC model, which was designed as a field scale model, to the gridded domain over the entire CONUS requires regional and county level parameterizations and soil data (Cooter et al., 2012). Further improvements in these parameterizations and data sets should reduce the frequency of unrealistic results.

## 5. Conclusions

The new implementation of ammonia bidirectional surface flux in the WRF-CMAQ modeling system is closely linked with the EPIC agricultural model through the FEST-C interface. The daily EPIC model simulated parameters including soil ammonia content, soil pH, soil moisture, and other soil characteristics (CEC, porosity, wilting point) are critical for calculating the soil emission potential, which defines the soil compensation concentration that is the primary source for the surface-to-air flux. EPIC is a comprehensive process-based agro-ecosystem model that has been successfully used worldwide (e.g., Edwards et al., 1994; Rosenzweig et al., 2014; Wriedt et al., 2009). The model is adapted to regional applications in FEST-C, and the system performs reasonably well given regional-management representations (Cooter et al., 2012). Thus, the coupled WRF-CMAQ-EPIC system is a significant advancement over other air quality models with bidirectional ammonia fluxes. Furthermore, with the system being linked with the hydrology and water quality SWAT model (Yuan et al., 2018), the WRF-CMAQ-EPIC-SWAT becomes a valuable tool for integrated multimedia modeling assessments of interactions among air-land-water processes.

The key components of the ammonia bidirectional flux model include the various resistance parameters that define the flux pathways to and from the soil and plant stomata and cuticle through the canopy. Many of these resistances are inherited from the dry deposition model in CMAQ (M3DRY; Pleim & Ran, 2011); some of which, aerodynamic resistance, boundary layer resistance, and stomatal resistance, are also used in the evapotranspiration model in WRF and then passed to CMAQ. The WRF-CMAQ system is designed to use the PX LSM, which features a data assimilation scheme that adjusts soil moisture and temperature to minimize errors in air temperature and humidity (Pleim & Gilliam, 2009; Pleim & Xiu, 2003). Therefore, parameters including soil moisture and temperature and the above-mentioned resistances that are all important to the bidirectional flux calculations are constrained by the data assimilation scheme and parameterized consistently. Other key resistances for the ammonia bidirectional flux model include soil resistance, in-canopy aerodynamic resistance, and cuticle resistance, all of which are quite uncertain and need further field experiment research. The uncertainties in these processes are not only in the parameterizations but also in the spatial heterogeneity of key quantities such as soil moisture, LAI, and canopy structure at the subgrid scale.

Overall, the coupled WRF-CMAQ-EPIC system with new bidirectional ammonia flux model is a significant improvement over the base methodology of prescribing fertilizer ammonia emissions from the NEI. Since direct measurements of NH_3_ fluxes over the CONUS domain are not available, evaluation of the model must be inferred from measurements of ambient concentrations of gas and aerosol concentrations and wet deposition. The combined analysis shows that the CMAQ bidi model well represents all three forms of ammonia with a relatively small negative bias. Given the uncertainties in many model parameters, these biases could easily switch to overestimation or larger underestimation with small adjustments to a few key parameters. Future research could investigate parameter sensitivities through combined parameter uncertainty experiments like sensitivities studies performed for PBL models (e.g., Nielsen-Gammon et al., 2010; Yang et al., 2017).

The combined evaluation also gives insight into other aspects of model performance such as the conclusion that the model may be underestimating wet scavenging of NH_3_ gas since NH_3_ concentrations are overestimated in the high emission areas near concentrated hog production while wet deposition of NH_4_^+^ is underestimated in these areas. Comparisons to CrIS retrievals are especially valuable for understanding the model’s mechanistic underpinnings. For example, the distinct pattern of high NH_3_ concentrations in the Central Valley, the Snake River Valley, and the western High Plains that is evident in the CrIS maps is also shown in the model maps and are all areas with high soil pH which results in high NH_3_ fluxes. The CrIS comparisons also demonstrate model limitations such as the underestimation of NH_3_ concentrations in the spring. Since the comparison of wet deposition against NADP NTN measurements also shows underestimation in the spring, it is likely that the model is underestimating the NH_3_ fluxes during these months.

Analysis of the 2016 WRF-CMAQ-EPIC simulations reveals some issues that cause unrealistically high fluxes and NH_3_ concentrations. High soil ammonia content along with high pH and dry soil results in extremely high *Γ*_*g*_ values that produce very large fluxes particularly in the Central Valley of CA. The anomalously high soil ammonia contents for particular crop types in some areas warrants further examination in the FEST-C EPIC system. As FEST-C EPIC is configured with regional-scale management representations, discrepancies at the local scale such as watershed, farms, and grid cells are expected. The large number of parameterizations inherent in the EPIC model and the extension to the CONUS domain understandably give sporadic consequential anomalies. As the system is further applied, evaluated, and refined in all components (WRF, CMAQ, EPIC) with improved land use, soil, and management representations at finer scales such errors will likely be reduced.

## Key Points:

- Bidirectional ammonia surface fluxes are modeled in an air quality model coupled to an agricultural ecosystem model
- Ammonia fluxes are strongly influenced by soil ammonia loading, soil pH, and soil moisture
- Satellite ammonia concentration retrievals show that the model simulates realistic monthly average ammonia concentrations
